# Identification of QTL-allele systems of seed size and oil content for simultaneous genomic improvement in Northeast China soybeans

**DOI:** 10.3389/fpls.2024.1483995

**Published:** 2024-11-14

**Authors:** Jianbo He, Lianshun Fu, Xiaoshuai Hao, Yicun Wu, Mengfan Wang, Qi Zhang, Weidan Feng, Mengmeng Fu, Yanping Wang, Haixiang Ren, Weiguang Du, Wubin Wang, Junyi Gai

**Affiliations:** ^1^ Soybean Research Institute & MARA National Center for Soybean Improvement & MARA Key Laboratory of Biology and Genetic Improvement of Soybean & State Key Laboratory of Crop Genetics and Germplasm Enhancement and Utilization & State Innovation Platform for Integrated Production and Education in Soybean Bio−Breeding & Zhongshan Biological Breeding Laboratory & Jiangsu Collaborative Innovation Center for Modern Crop Production, Nanjing Agricultural University, Nanjing, China; ^2^ Soybean Research Institute, Tieling Academy of Agricultural Sciences, Tieling, China; ^3^ Mudanjiang Research and Development Center for Soybean & Mudanjiang Experiment Station of the National Center for Soybean Improvement, Mudanjiang Branch of Heilongjiang Academy of Agricultural Sciences, Mudanjiang, China

**Keywords:** Northeast China soybean germplasm population (NECSGP), 100-seed weight (100SW), seed oil content (SOC), RTM-GWAS, recombination potential prediction, simultaneous genomic cross design for traits, evolutionary dynamics

## Abstract

Northeast China (NEC) is the major production area for soybeans in China, whereas its soybean germplasm has played key roles in world soybean production, especially in the Americas. For plant breeding, genomic selection involves two stages, cross design and progeny selection, with the former determining the latter’s potential. In NEC, one of the major breeding purposes is for 100-seed weight (100SW) and seed oil content (SOC). A diverse sample with 361 NEC soybean germplasm accessions was evaluated for their 100SW and SOC in Tieling, Liaoning, China. Both traits exhibited significant phenotypic, genotypic, and G × E variation, with a trait heritability of 82.38% and 86.26%, respectively. A restricted two-stage multi-locus genome-wide association study (RTM-GWAS) with 15,501 SNPLDB (SNP linkage disequilibrium block) markers identified 80 and 92 QTLs with 230 and 299 alleles for 100SW and SOC, respectively. Corresponding to some increase of the two traits, almost all the alleles in the early maturity groups (MG 0 + 00 + 000) were inherited from the late MGs (MG I+II+III), indicating that genetic recombination was the major motivator in addition to a few allele emergence and some allele exclusion fluctuations among early MGs. Using the 95th percentile as indicator, the prediction of recombination potentials showed that 30.43 g 100SW and 27.73% SOC might be achieved, respectively. Three strategies of simultaneous genomic improvement of both traits in designing optimal crosses, namely, 100SW-first, SOC-first, and 100SW-SOC-balance, were proved to be efficient. Thus, the optimal cross design could be extended to multiple traits based on a relatively thorough identification of the QTL-alleles using RTM-GWAS.

## 1 Introduction

Northeast China (NEC) is the major production area for soybeans, with its yield and acreage accounting for approximately 50% in China. Liu et al. (2020) showed that soybeans in North and South America were genetically clustered in the same group with those from NEC, and the germplasm from NEC is the primary source for soybeans in the world’s largest production regions. Therefore, exploring the genetic basis of NEC soybean germplasm is essential for global soybean production (Fu et al., 2020a). During the long period of artificial selection and improvement in soybean, the seed size (expressed as 100-seed weight or 100SW), seed oil content (SOC), and seed protein content (SPC) increased, but there was a negative correlation between oil and protein contents (Guo et al., 2022; Li et al., 2022; Ray et al., 2022). Compared with soybeans in central and southern China, soybeans in NEC have relatively smaller 100SW but higher SOC; thus, one of the major breeding purposes is for increased 100SW and SOC.

Marker-assisted selection (MAS) has been proven as an effective method for precise plant breeding (Collard and Mackill, 2008). Through identifying genomic markers related to breeding targets, MAS improves breeding efficiency by applying directly genotypic selection in addition to phenotypic selection. Furthermore, the concept of “breeding by design” was proposed based on quantitative trait locus (QTL) and gene mapping, aiming to improve breeding efficiency by designing optimal genotypes and parental combinations (Peleman and van der Voort, 2003). However, breeding by design is usually applicable for the selection of a handful of major genes. Meanwhile, genomic selection (GS) was also proposed to improve breeding efficiency by predicting breeding values in offspring population using whole-genome markers without the need for QTL/gene mapping (Meuwissen et al., 2001). GS firstly establishes a statistical model between phenotype and genome-wide markers in a training or reference population (generally germplasm population) where both phenotype and genotype data are available and then predicts breeding values as a comprehensive evaluation for all target traits in breeding populations using genome-wide markers based on an established model. In fact, both breeding by design and GS are special cases of MAS in comprehensive selection for multiple traits. Following the “breeding by design” concept, QTL-allele-based GS was also proposed as a potential approach to both optimal crosses and superior progenies based on the whole-genome QTL-allele system, and is in fact a direct genotype selection method (He et al., 2017). Anyway, simultaneous improvement of multiple traits using QTL-allele-based GS in plant breeding needs to be further explored and practiced.

Both 100SW and SOC are quantitatively inherited traits controlled by a number of genes with varying effects. At least 297 and 315 QTLs have been reported at SoyBase (https://www.soybase.org) for 100SW and SOC in soybean, respectively. These QTLs were detected mainly from bi-parental populations using the linkage mapping method with many ones located in overlapping regions. However, among these, only 13 and 16 QTLs of 100SW and SOC were recognized as confirmed QTLs, respectively (Fasoula et al., 2004; Nichols et al., 2006; Pathan et al., 2013). There were also a handful of genes conferring the two traits, which have been cloned. For example, a wild soybean allele simultaneously conferring 100SW, seed protein, and oil content was mapped to a 329-kb region on chromosome 15, with *Glyma.15g049200* as their common candidate gene (Yang et al., 2019). The causative gene *SW16.1* underlying a 100SW QTL *qSW16.1* was identified and encoded as a nucleus-localized LIM domain-containing protein (Chen et al., 2023). However, the underlying genes for most of the detected QTLs still need to be further identified; in particular, the complete gene systems of 100SW and SOC QTLs need to be explored for a thorough genetic design.

The linkage mapping for QTL detection involves only two parental lines, such as the recombinant inbred line (RIL) population, in which the genetic variation is limited between the two parents. The genome-wide association study (GWAS) for the natural/germplasm population provides a broad background to genome-wide QTL identification (Hong et al., 2023; Khatun et al., 2022; Ren et al., 2022; Ullah Zaid et al., 2018) and was widely used in soybean for QTL identification (Li et al., 2017; Zhang et al., 2018; Su et al., 2023). The GWAS on over 12,000 soybean accessions identified 18 and 19 QTLs for SOC and SPC, respectively (Bandillo et al., 2015). Based on 809 soybean accessions worldwide, GWAS identified 245 QTLs of 84 agronomic traits (Fang et al., 2017). Hwang et al. (2014) identified 25 and 40 SOC and SPC loci in 298 soybean germplasm accessions, respectively. The GWAS also provided an efficient method for mining the underlying genes conferring quantitative trait variation. A sucrose efflux transporter gene, *GmSWEET39*, controlling SOC was identified using GWAS (Miao et al., 2020). A pair of *SWEET* homologs, *GmSWEET10a* and *GmSWEET10b*, were identified simultaneously conferring 100SW, SOC, and SPC during soybean domestication (Wang S. et al., 2020).

Generally, only a handful of major QTLs were detected in individual GWAS, whereas their multiple alleles in germplasm populations were neglected. To improve the GWAS efficiency, the restricted two-stage multi-locus genome-wide association analysis (RTM-GWAS) was proposed for a relatively thorough detection of whole-genome QTLs with their multiple alleles (He et al., 2017). Two innovative techniques in RTM-GWAS were taken. One method uses SNPLDB (SNP linkage disequilibrium block) markers with multiple haplotypes to meet the requirements of multiple alleles in the natural population. The other method controls the total contribution within heritability value based on two-stage GWAS with the first stage under a single-locus model for marker pre-selection and the second stage of stepwise regression under a multi-locus model with forward addition and backward elimination. RTM-GWAS was used in QTL-allele detection for 100SW and SOC in 366 soybean landraces (Zhang et al., 2015, 2018). Firstly, 116,769 single-nucleotide polymorphism (SNP) markers were used to form 29,121 SNPLDBs. Then, 55 and 50 QTLs with 263 and 136 alleles were detected based on SNPLDBs for 100SW and SOC, respectively. The detected QTL-allele matrix was used in studies for evolutionary motivators and breeding potentials of the respective trait.

Conventional crossbreeding has been the major procedure for genetic improvement in soybean. It consists of two major steps: the first is to design optimal crosses based on germplasm or breeding materials, and the second is to select the best progenies in segregating generations. Optimal cross design determines the potential of progeny selection and is the key to breakthrough breeding. An approach of GS based on the QTL-allele matrix was proposed for optimal cross design (He et al., 2017; He and Gai, 2020) and has been applied to soybean breeding (Khan et al., 2018; Wang W. et al., 2020; Fahim et al., 2021; Wang et al., 2021). For example, 1,803 optimal crosses were predicted for high SPC in the NEC soybean germplasm population (NECSGP), and the maximum of predicted SPC was 50.00% with a transgressive potential of 3.93% improvement (Feng et al., 2022).

However, previous studies on cross design based on GWAS-identified QTL-allele results focused on genetic improvement of a single trait. Since breeding programs involve multiple traits, it is essentially appropriate to consider multiple traits simultaneously in designing optimal crosses. In this study, the representative sample of the NECSGP (Feng et al., 2022) was studied to identify the 100SW and SOC QTL-allele systems expressed in NEC using the RTM-GWAS procedure, to characterize the genetic systems and motivators of the two traits in the evolutionary process from the southern part to the northern part of NEC, and to predict the recombination potential or to design optimal crosses for the simultaneous improvement of the two traits in the NECSGP using 100SW-first, SOC-first, and 100SW-SOC-balance strategies, respectively. This optimal cross design method could be extended to multiple traits based on a relatively thorough identification of the QTLs and their alleles of the traits.

## 2 Materials and methods

### 2.1 Plant materials and field experiments

As described previously (Feng et al., 2022), a total of 361 soybean accessions from the NEC were used in this study. These accessions involve six soybean maturity groups, namely, III, II, I, 0, 00, and 000 (Fu et al., 2020a). Accessions in MG III matured later, whereas accessions in MG 000 matured earlier. Field experiments were performed at Tieling, Liaoning in 2013–2014. All accessions were grouped into six blocks according to maturity group and were planted using the “blocks in replication” design with four replications. Normal field management including weed control and fertilization was used. The matured seeds were harvested and dried under 35–40°C. The 100SW (g) was measured on 100 randomly selected seeds, and the SOC was determined by the near-infrared grain analyzer Infratec 1241 (FOSS, Hilleroed, Denmark).

### 2.2 Statistical analysis

Statistical analysis was performed using the SAS Studio software through SAS OnDemand for Academics (https://welcome.oda.sas.com/). Joint analysis of variance (ANOVA) was conducted using the PROC GLM procedure, while the variance components were calculated using PROC VARCOMP in which genotype, environment, replication within environment, and genotype × environment were considered as random effects. The heritability (*h* ^2^) was estimated as $h^{2}=\sigma_{ɡ}^{2}/\left(\sigma_{ɡ}^{2}+\sigma^{2}/r\right)$ for the single environments and $h^{2}=\sigma_{ɡ}^{2}/\left(\sigma_{ɡ}^{2}+\sigma_{ɡt}^{2}/t+\sigma^{2}/tr\right)$ for the combined data over multiple environments, where $\sigma_{ɡ}^{2}$ , $\sigma_{ɡt}^{2}\;$ and $\sigma^{2}$ are variance of the genotype, genotype × environment, and random error; *t* is the number of environments; and *r* is the number of replications (Hanson et al., 1956).

### 2.3 Genotyping and haplotype block marker construction

The genotype data of the 361 accessions were obtained from Fu et al. (2020a). Whole-genome sequencing of the accessions were carried out using RAD-seq (restriction-site associated DNA sequencing) at BGI Tech, Shenzhen, China. The genomic DNA was isolated from the young leaves of soybean seedlings according to the conventional CTAB method (Murray and Thompson, 1980). Paired-end sequencing was conducted on an Illumina HiSeq2000 platform through the multiplexed shotgun genotyping method (Andolfatto et al., 2011). All sequence reads were aligned against the genome of Williams 82 using the SOAP2 software (Li et al., 2009; Schmutz et al., 2010). RealSFS (Yi et al., 2010) was utilized to detect SNP loci, which were filtered with a maximum missing and heterozygous allele call rate of ≤20% and a minimum minor allele frequency (MAF) of ≥1%. The fastPHASE software (Scheet and Stephens, 2006) was used for genotyping the SNP imputation resulting in 82,966 high-quality SNPs. The SNPs after quality control were grouped into 15,501 SNPLDBs based on the LD threshold of *D*’ ≥0.7 according to He et al. (2017), while haplotypes were treated as alleles of a QTL.

### 2.4 Detection of the QTL-allele system and the establishment of matrix/sub-matrices

The RTM-GWAS software (https://gitee.com/njau-sri/rtm-gwas) was used to detect the QTL-allele system of 100SW and SOC in the NECSGP. The genetic similarity between accessions was estimated based on genome-wide SNPLDBs, and the top 10 eigenvectors of the genetic similarity coefficient matrix were used as the covariates to correct the population structure bias. A threshold of *p* = 0.05 was used at the first stage of RTM-GWAS for candidate marker preselection, and a significance level of *p* = 0.05 was used for QTL detection through stepwise regression at the second stage of RTM-GWAS. The detected QTLs (associated SNPLDBs) with their allele effects for each accession were used to establish the QTL-allele matrix, which was further separated into maturity group sub-matrices for further analysis on evolutionary motivators.

### 2.5 Candidate gene annotation

The candidate genes for 100SW and SOC from the detected QTLs were annotated through the following steps: (1) the genes of soybean genome Wm82.a1.v1.1 within the genomic interval of a detected QTL (with a 50-kb flanking expansion) were retrieved from SoyBase (https://www.soybase.org); (2) the independence of SNP(s) between an identified SNPLDB and gene(s) within the genomic interval was statistically tested using the chi-square criterion at a significance level of 0.05; and (3) the Gene Ontology (GO) annotations of significantly correlated genes were retrieved from SoyBase (https://www.soybase.org).

### 2.6 Recombination potential prediction and optimal cross design

All possible 64,980 crosses (361×360/2) were generated *in silico*. For each cross, the genotype data of 2,000 homozygous progenies were simulated through continuous selfing starting from F_2_ generation under the linkage model, where the number of crossovers on each chromosome was simulated randomly according to the Poisson distribution with chromosome length as a parameter. The predicted genotypic values of each progeny were calculated as the sum of all allele effects plus the population mean (He et al., 2017). Different percentiles of the progeny population were calculated and used as the predicted recombination potential. The *cross* program (https://gitee.com/njau-sri/cross) was used for simulation. In addition, the crosses were also grouped according to maturity groups, then the recombination potential within and among maturity groups was also analyzed.

Three strategies were proposed in this study for two-trait optimal cross design of soybean with both high 100SW and SOC. In the first strategy (designated 100SW-first), the top 100 crosses with the highest recombination potential for 100SW were selected firstly, and then the top 10 crosses with the highest recombination potential for SOC were selected from the 100 crosses. In the second strategy (designated SOC-first), the top 100 crosses with the highest recombination potential for SOC were selected firstly, and then the top 10 crosses with the highest recombination potential for 100SW were selected from the 100 crosses. The third strategy (designated 100SW-SOC-balance) involved balanced selection for 100SW and SOC; firstly, all possible crosses were arranged in descending order according to the recombination potential for 100SW and SOC, respectively, and then the top 10 most common crosses were selected from the top 2,000 100SW and SOC crosses.

## 3 Results

### 3.1 Variation of 100SW and SOC in the NECSGP

**Figure 1:**
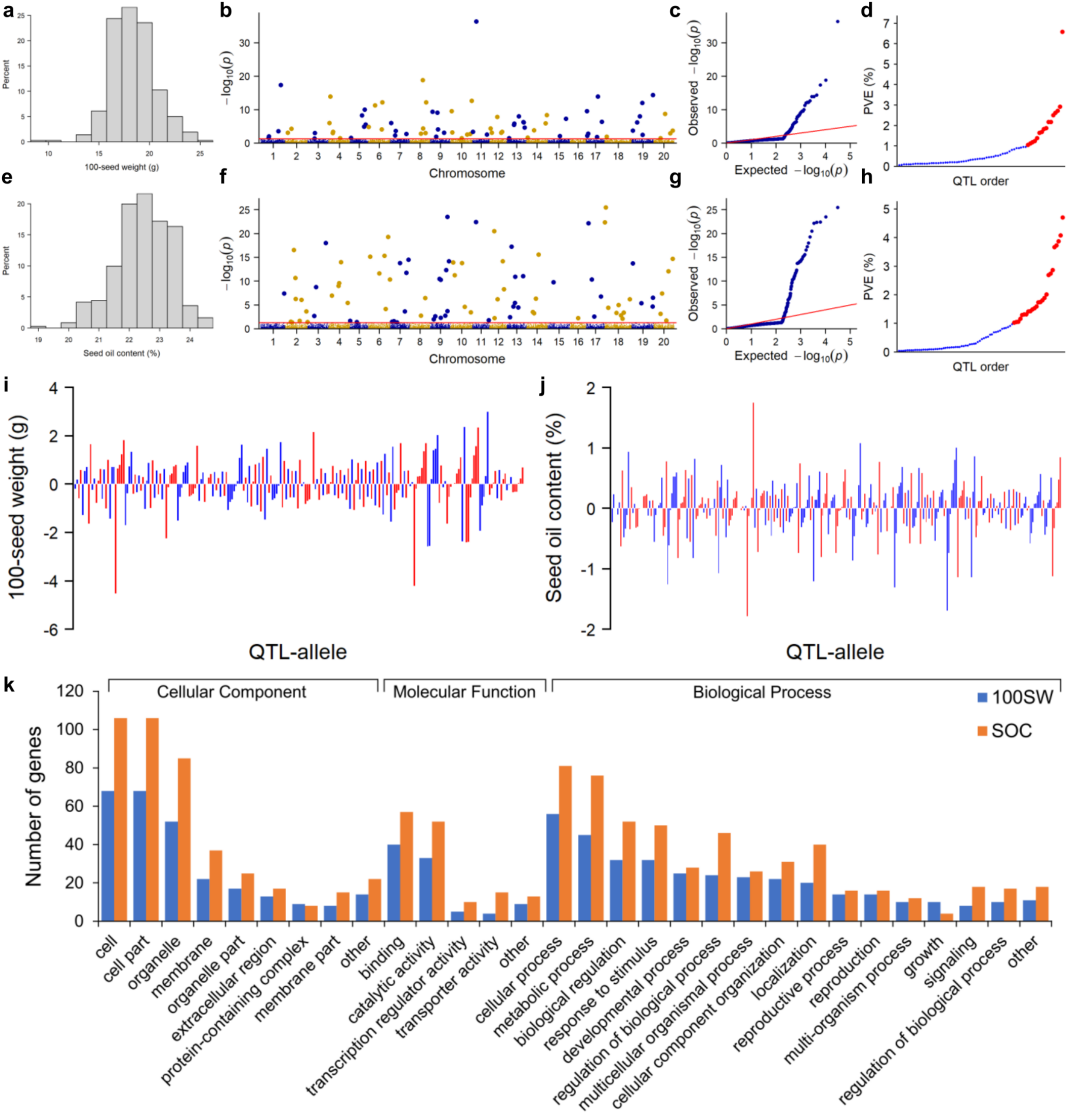
QTL-allele detection for 100-seed weight and seed oil content in the NECSGP. **(A–D)** Genome-wide association study of 100-seed weight: histogram of phenotype data **(A)**, Manhattan plot **(B)** and quantile–quantile plot **(C)** of marker *p*-values, and phenotypic contribution (*R* ^2^) of the main-effect QTLs in ascending order **(D)** with blue color for small-contribution QTL (*R* ^2^ < 1%) and red color for large-contribution QTL (*R* ^2^ ≥ 1%). **(E–H)** Genome-wide association study of seed oil content: histogram of phenotype data **(E)**, Manhattan plot **(F)** and quantile–quantile plot **(G)** of marker *p*-values, and phenotypic contribution (*R* ^2^) of the main-effect QTLs in ascending order **(H)** with blue color for small-contribution QTL (*R* ^2^ < 1%) and red color for large-contribution QTL (*R* ^2^ ≥ 1%). **(I)** Allele effects of main-effect QTLs for 100SW. **(J)** Allele effects of main-effect QTLs for SOC. **(K)** Candidate gene annotation for 100SW and SOC QTLs.

The joint ANOVA over 2 years indicated that the 100SW and SOC varied both significantly among accessions (genotypes), as well as their genotype × environment interactions (GEI, **Supplementary Table 1**) in the NECSGP. However, the estimated GEI variances were relatively small compared to the genotypic variance, indicating that the interaction between genotype and environment (year) was weak for the two traits in the NECSGP. Both 100SW and SOC exhibited higher heritability values over two environments, 82.46% and 86.34, respectively. The 100SW in the NECSGP ranged from 9.00 to 27.20 g across 2 years, with an average of 18.37 g (**Table 1**; **Figure 1A**), which was not as wide as that in the Chinese soybean landrace population (CSLP, ranging from 4.59 to 40.35 g in Zhang et al., 2015). The SOC in the NECSGP ranged from 18.80% to 24.85% across 2 years, with an average of 22.45% (**Table 1**; **Figure 1E**), which was similar to the CSLP (ranging from 14.95% to 26.42%) in Zhang et al. (2018). The genetic coefficient of variation (*GCV*) of SOC varied relatively less (4.12%) than that of 100SW (11.77%) (**Table 1**). The above results indicate that the variability of 100SW and SOC in the NECSGP is not better than those of CSLP; the improvement of the two traits needs to explore their recombination potential.

**Table 1:** The distribution and descriptive statistics of 100-seed weight and seed oil content in the NECSGP.

Trait	Year	Midpoint													*N*	Mean	Range	*GCV* (%)	*h* ^2^ (%)
100SW (g)		9.0	10.5	12.0	13.5	15.0	16.5	18.0	19.5	21.0	22.5	24.0	25.5	27.0					
	2013	1	1	1	2	8	57	90	83	66	30	14	6	2	361	19.31	9.18–27.20	12.50	87.08
	2014	1	1	2	22	45	107	86	55	27	10	5			361	17.44	9.00–24.63	13.06	79.67
	Mean	1	1	0	5	22	87	97	84	38	18	7	1		361	18.37	9.09–25.16	11.77	82.38
SOC (%)		19.0	19.5	20.0	20.5	21.0	21.5	22.0	22.5	23.0	23.5	24.0	24.5	25.0					
	2013	1	5	7	21	16	38	60	64	74	46	21	7	1	361	22.39	18.80–24.75	4.79	92.41
	2014		2	1	12	15	37	58	88	70	42	27	4	1	357	22.52	19.55–24.85	3.99	92.25
	Mean	1	0	3	15	16	37	71	78	62	59	13	6		361	22.45	19.21–24.73	4.12	86.26

100SW, 100-seed weight; SOC, seed oil content. *N*, the number of accessions. *GCV*, the genetic coefficient of variation defined as genetic standard deviation divided by phenotype mean. *h* ^2^, trait heritability. The SOC was evaluated with the near-infrared grain analyzer Infratec 1241 (FOSS, Hilleroed, Denmark), and its value might be inflated at some degree. However, all the evaluation of SOC was kept under the same environment and comparable.

There were some variations of 100SW and SOC among different maturity groups in the NECSGP, but the major difference was within maturity groups. Overall, the 100SW and SOC in the late maturity groups (MG I+II, except for III for the former, and MG II+III, except for I for the latter) were smaller than those in early maturity groups (MG 000 + 00 + 0) (**Supplementary Table 2**). This suggested that both 100SW and SOC had been genetically improved in the NECSGP when adapting to high latitude with days to maturity shortened.

The top 10 100SW accessions (F67, F326, F406, F147, F58, P004, P085, F36, P188, and F315 in descending order) ranged from 25.16 to 23.05 g (average for 2 years), with their SOC ranging from 22.60% to 20.38%. On the other hand, the top 10 SOC accessions (F82, F386, F135, F79, F32, F155, F53, F305, F306, and F351 in descending order) ranged from 24.73% to 24.09% (average for 2 years), with their 100SW ranging from 21.04% to 15.64%. No accession had both the highest 100SW and the highest SOC in the NECSGP. The correlation analysis showed that 100SW and SOC did not exhibit a significant Pearson correlation coefficient (*r* = 0.008, *p* = 0.8722), suggesting that simultaneous improvement of seed size and oil content might be possible in the NECSGP. To effectively improve both 100SW and SOC in soybean breeding, a thorough detection of the whole-genome QTL-allele constitution for these two traits is an essential requirement.

### 3.2 Genome-wide detection of 100SW and SOC QTL-allele systems in the NECSGP

As described previously in Feng et al. (2022), a total of 15,501 SNPLDBs were constructed from 82,966 SNPs in a RAD-seq procedure in the NECSGP. In the first stage of RTM-GWAS under the single-locus model, 8,272 and 8,863 out of 15,501 SNPLDBs were preselected for 100SW and SOC, respectively. In the second stage under the multi-locus model, 80 and 92 QTLs were detected for 100SW and SOC, respectively (**Figures 1B, C, F, G**).

For 100SW, 80 QTLs were identified (**Supplementary Table 3**; **Figure 1D**). These QTLs were distributed on 19 chromosomes except for chromosome 16, with seven QTLs located on chromosome 13 being the highest. Among the 80 QTLs, 42 had the main effect only, 8 QTLs had the QEI effect only, and 30 QTLs had both the main effect and QEI effect. The phenotypic contribution of the 72 QTLs with a significant main effect varied continuously from 0.06% to 6.58% in a total of 54.11% phenotypic variation (**Table 2**). The 16 large-contribution QTLs (main effect *R* ^2^ ≥1%, around the inflection point of the phenotypic contribution curve) explained 34.60% phenotypic variation, and 56 small-contribution QTLs (main effect *R* ^2^ <1%) explained 19.51% phenotypic variation. A total of 230 alleles were identified on the 80 QTLs, with the number of alleles per QTL ranging from 2 to 8, and 27 QTLs had at least 3 alleles (**Figure 1I**).

**Table 2:** Summary of the QTL-allele system for 100-seed weight and seed oil content in the NECSGP.

QTL-allele	100-seed weight		Seed oil content	
	Main effect	QTL×Year	Main effect	QTL×Year
QTL (*R* ^2^, %)	54.11 (72, 0.06–6.58)	7.34 (38, 0.08–0.53)	70.06 (82,0.04–4.70)	9.08 (54, 0.03–0.75)
LC QTL (*R* ^2^, %)	34.60 (16, 1.05–6.58)		53.10 (25, 1.02–4.70)	
SC QTL (*R* ^2^, %)	19.51 (56, 0.06–0.95)	7.34 (38, 0.08–0.53)	16.96 (57, 0.04–0.97)	9.08 (54, 0.03–0.75)
Unmapped QTL (*R* ^2^, %)	28.35	0.58	16.28	0.27
Heritability (*h* ^2^, %)	82.46	7.92	86.34	9.35
Total allele	232 (–4.53–3.00)	464 (–1.75–1.75)	299 (–1.79–1.75)	598 (–1.11–1.11)
Positive allele	121 (0.00–3.00)	232 (0.00–1.75)	161 (0.00–1.75)	299 (0.00–1.11)
Negative allele	111 (–4.53–0.04)	232 (–1.75–0.00)	138 (–1.79–0.00)	299 (–1.11–0.00)

Main effect: main-effect QTL. QTL×Year: QTL by Year interaction. *R* ^2^: phenotypic variation explained. LC QTL: large-contribution QTL (*R* ^2^ ≥ 1%). SC QTL: small-contribution QTL (*R* ^2^ < 1%). In parentheses of QTL rows, the first number is the number of identified QTLs, followed by a range of single QTL contributions to phenotypic variance. In parentheses of allele rows is the range of single allele effects.

For SOC, 92 QTLs were identified (**Supplementary Table 4**; **Figure 1H**). These QTLs were distributed on all 20 chromosomes, with 10 QTLs on chromosome 9 being the highest. Among the 92 QTLs, 38 had the main effect only, 10 QTLs had only the QEI effect, and 44 QTLs had both the main effect and QEI effect. Thus, 82 QTLs had significant main effects that varied continuously from 0.04% to 4.70% in a total of 70.07% phenotypic variation. The 25 large-contribution QTLs explained 53.10% and 57 small-contribution QTLs explained 16.96% phenotypic variation (**Table 2**). There were 299 alleles on the 92 QTLs, with the number of alleles per QTL ranging from 2 to 8, and 38 QTLs with 3 or more alleles (**Figure 1J**).

**Figure 2:**
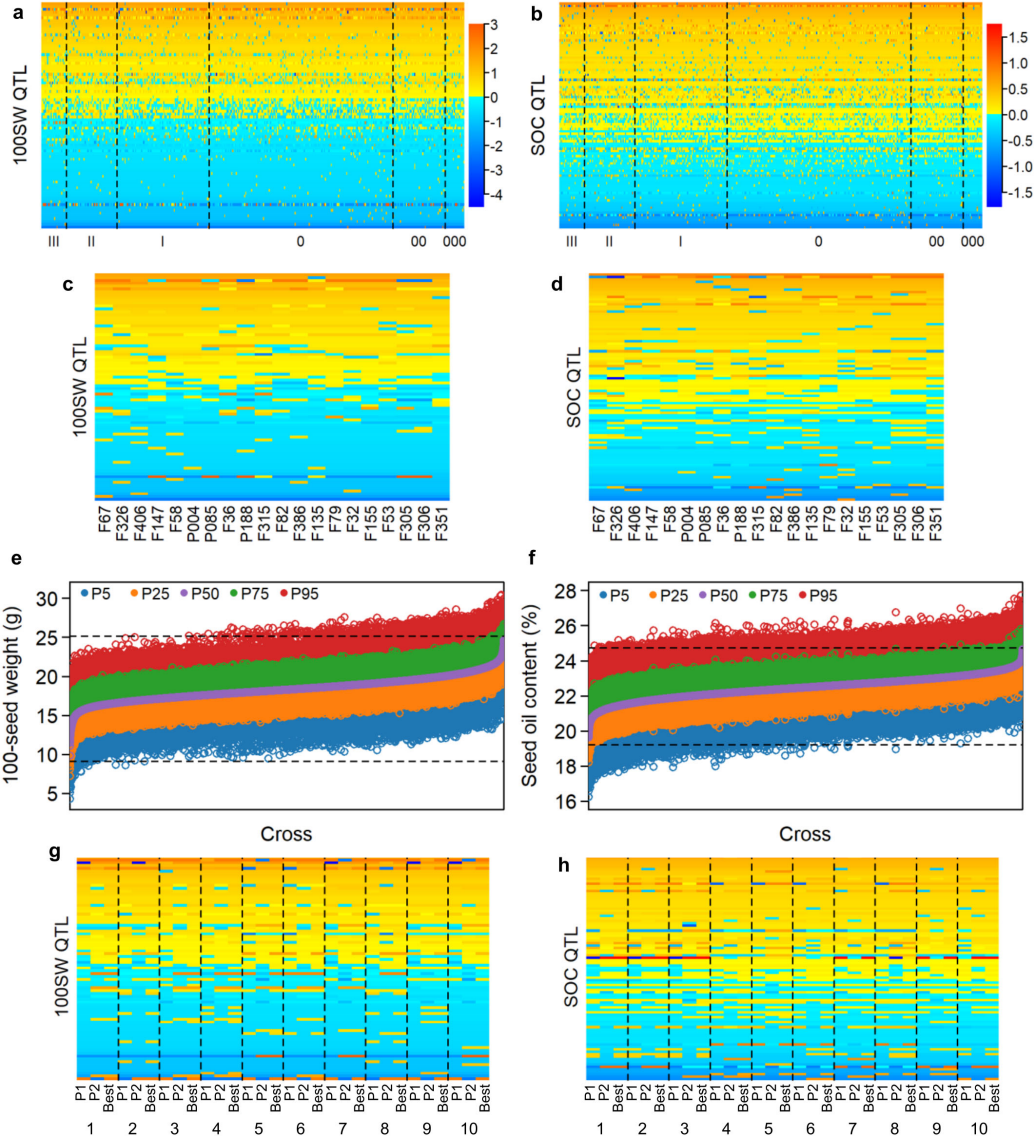
QTL-allele matrix and recombination potential of 100-seed weight and seed oil content in the NECSGP. **(A)** Graphical representation of the QTL-allele matrix of 100SW. The horizontal axis represents accessions, while the vertical axis represents QTL arranged in a rising order of their positive allele frequency. Each row represents the allele distribution among accessions for a QTL, while each column indicates the allele constitution of an accession over all QTLs. Allele effects are expressed in color cells with warm colors indicating positive effects and cool colors indicating negative effects, and the color depth indicates effect size. **(B)** Graphical representation of the QTL-allele matrix of SOC. **(C)** The QTL-allele matrix of 100SW for the 20 best accessions. **(D)** The QTL-allele matrix of SOC for the 20 best accessions. **(E)** Distribution of predicted 100SW of progenies in all possible crosses, with the maximum and minimum (upper and lower horizontal dotted lines). **(F)** Distribution of predicted SOC of progenies in all possible crosses, with the maximum and minimum (upper and lower horizontal dotted lines). **(G)** Graphical representation of the QTL-allele matrix of 100SW for the top 10 optimal crosses (1–10 in **Supplementary Table 5**) each with P1, P2, and the best progeny. **(H)** Graphical representation of the QTL-allele matrix of SOC for the top 10 optimal crosses (1–10 in **Supplementary Table 6**) each with P1, P2, and the best progeny.

The allele effect of main effect QTLs ranged from −4.53 to 3.00 g for 100SW and from −1.79 to 1.75% for SOC (**Figures 1I, J**). The 72 and 82 main effect QTLs and their allele effects for each of the 361 accessions were organized as a QTL-allele matrix, respectively (**Figures 2A, B**), a compact form of the genetic constitution of the two traits in the NECSGP. In addition, the QTL-allele matrix was further separated into sub-matrices corresponding to the six maturity groups for comparisons and evolutionary changes of 100SW and SOC among maturity groups.

The QTL-allele constitution of the top 10 accessions of the highest 100SW and SOC is shown in **Figures 2C, D**, respectively. These accessions contained many alleles of negative effects on most QTLs, indicating large improvement potential for both 100SW and SOC.

In the following text, the focus will be on the main effect QTL-allele matrices, while the GEI matrices will be left for future analysis, as the environment factor of year varied not definitely but randomly.

### 3.3 Annotation of the candidate gene system of 100SW and SOC in the NECSGP

Among the detected 100SW QTLs, 87 genes were annotated from 37 QTLs, while no candidate genes were annotated for the remaining 43 QTLs, according to SoyBase (https://www.soybase.org). These 87 genes were distributed on 17 chromosomes, excluding Gm01, Gm08, and Gm16. Similarly, 132 genes were annotated from 40 SOC QTLs, while no candidate genes were annotated for the remaining 52 SOC QTLs. These 132 genes were distributed on 16 chromosomes, excluding Gm01, Gm05, Gm08, and Gm16. GO annotations indicated that these genes involved cellular components, molecular functions, and biological processes, especially the latter, including 16 function groups (**Figure 1K**). This suggested that the candidate gene systems of the two traits involved multiple different groups of functions. The QTL *qSW-11-2* with the highest explained phenotypic contribution to 100SW (*R* ^2^ = 6.58) had nine associated genes, among which *Glyma11g12120* (vacuolar proton ATPase A3) and *Glyma11g12230* (clathrin adaptor complexes medium subunit family protein) were highly expressed in soybean pod and seed (SoyBase, http://www.soybase.org). In the SOC QTL *qSOC-18-2* with the highest explained phenotypic variation (*R* ^2^ = 4.7%), three genes were annotated including *Glyma18g03450*, a HAD superfamily phosphatase highly expressed in soybean pod and seed. In addition, based on the gene models for which there was a significant change of gene expression in seed developmental stages (Severin et al., 2010), three genes (*Glyma09g25490*, *Glyma10g05580*, and *Glyma13g36780*) and four genes (*Glyma07g32050*, *Glyma09g32980*, *Glyma11g10600*, and *Glyma19g42380*) were identified from the annotated genes for 100SW and SOC, respectively. In particular, two genes, *Glyma09g32980* and *Glyma11g10600*, were annotated from two large-contribution SOC QTLs (*qSOC-9-8* with *R* ^2^ = 4.07 and *qSOC-11-1* with *R* ^2^ = 3.87). These findings provide insights into the genetic architecture underlying 100SW and SOC and highlight specific genes that may be important for these traits.

### 3.4 Genetic differentiation of QTL-allele from late to early maturity groups

During the evolutionary process from the late (MG I+II+III) to early (MG 0 + 00 + 000) maturity groups, all 225 alleles for 100SW and 292 out of 294 alleles for SOC were retained (**Table 3**), suggesting that inheritance or migration was the major genetic dynamics for these two traits. Seven positive effect alleles emerged in the early maturity groups for 100SW. Two negative and three positive alleles emerged, and one negative and one positive allele were excluded in the early maturity groups for SOC. Between MG 0 + 00 + 000 and MG I+II+III, only a few allele changes happened for the two traits. However, the QTL-allele structure of both 100SW and SOC exhibited genetic fluctuation among the three early maturity groups, particularly with a large number of excluded alleles. Among the early maturity groups, MG 000 inherited only 156 100SW alleles and 186 SOC alleles from the late maturity groups (MG I+II+III), with 30% (69/225) of the 100SW alleles and 37% (108/294) of the SOC alleles excluded, while newly emerged alleles increased very little. In this case, for the two major genetic dynamics, allele emergence was quite limited whereas allele exclusion fluctuated; therefore, genetic recombination among alleles on different QTLs might play an important role in generating phenotypic variation among maturity groups. This might be the major genetic basis of the predicted transgressive optimal crosses with linkage obstacles deleted.

**Table 3:** The QTL-allele changes of 100 seed weight and seed oil content among maturity groups.

Trait	Maturity group	Total		Inherent		Emerged		Excluded	
	Contrast	Allele	QTL	Allele	QTL	Allele	QTL	Allele	QTL
100SW	I+II+III	225 (111,114)	80						
	0 vs. I+II+III	226 (109,117)	80	219 (109,110)	80	7 (0,7)	7	6 (2,4)	6
	00 vs. I+II+III	196 (92,104)	80	190 (92,98)	80	6 (0,6)	6	35 (19,16)	27
	000 vs. I+II+III	156 (72,84)	80	156 (72,84)	80	0 (0,0)	0	69 (39,30)	52
	0 + 00 + 000 vs. I+II+III	232 (111,121)	80	225 (111,114)	80	7 (0,7)	7	0 (0,0)	0
SOC	I+II+III	294 (136,158)	92						
	0 vs. I+II+III	293 (133,160)	92	288 (131,157)	92	5 (2,3)	4	6 (5,1)	5
	00 vs. I+II+III	239 (97,142)	92	235 (96,139)	92	4 (1,3)	4	59 (40,19)	51
	000 vs. I+II+III	188 (81,107)	92	186 (81,105)	92	2 (0,2)	2	108 (55,53)	74
	0 + 00 + 000 vs. I+II+III	297 (137,160)	92	292 (135,157)	92	5 (2,3)	4	2 (1,1)	2

100SW, 100-seed weight; SOC, seed oil content. The number outside parentheses is the total of alleles; the numbers in parentheses are the number of negative effect alleles and positive effect alleles, respectively. Inherent allele means alleles passed from the compared maturity group(s); Emerged allele means the alleles new to the compared maturity group(s); Excluded allele means the alleles excluded in the maturity group(s).

### 3.5 Optimal cross design for the improvement of individual and simultaneous improvement of 100SW and SOC in the NECSGP

The genetic recombination potential for 100SW and SOC in the NECSGP was predicted based on their respective QTL-allele matrix. As the breeding target is high 100SW or SOC, the 95th percentile of predicted 100SW or SOC in the progeny population was used as an indicator for the recombination potential of a parental cross in the NECSGP. As shown in **Table 4** and **Figures 2E, F**, the transgressive potential of the progenies for all possible 64,980 crosses among the 361 accessions was predicted for the respective traits in the NECSGP. For 100SW, the average recombination potential was 21.62 g, reaching a maximum of 30.42 g. This surpassed the observed 100SW in the NECSGP, which had a mean of 18.37 g and a maximum of 25.16 g, leading to a maximum improvement. Similarly, the average recombination potential for SOC was 24.42%, reaching a maximum of 27.73%, surpassing the observed average (22.45%) and maximum (24.73%) in the NECSGP.

**Table 4:** Predicted 95th percentile of 100SW and SOC of all possible crosses in/between maturity groups in the NECSGP.

Maturity group	*N*	100-seed weight (g)			Seed oil content (%)		
		Min.	Max.	Mean	Min.	Max.	Mean
Entire	64,980	15.76	30.43	21.62	21.32	27.73	24.20
I+II+III	10,153	16.03	29.54	21.21	21.32	27.14	23.94
0 + 00 + 000	23,653	16.72	28.57	21.84	21.81	27.12	24.35
0	12,246	16.48	29.52	21.68	22.07	27.12	24.37
00	990	17.48	27.74	22.38	22.16	26.44	24.39
000	120	18.89	26.31	21.90	22.44	25.07	23.93
0 + 00 + 000 vs. I+II+III	31,174	15.76	30.43	21.59	21.54	27.73	24.17
0 vs. I+II+III	22,451	15.98	30.43	21.49	21.54	27.42	24.18
00 vs. I+II+III	6,435	15.76	29.99	21.89	22.16	27.73	24.21
000 vs. I+II+III	2,288	17.52	29.27	21.67	21.78	26.73	23.98
0 vs.00	7,065	16.58	28.87	22.04	21.99	27.10	24.40
0 vs. 000	2,512	17.66	28.62	21.79	21.81	26.41	24.16
00 vs. 000	720	18.08	27.93	22.11	22.12	26.42	24.15

*N*: The number of crosses. SD, standard deviation. The predicted phenotypic value for each cross was defined as the 95th percentile of the predicted progeny. 0 + 00 + 000 vs. I+II+III means crosses between 0 + 00 + 000 and I+II+III, and the same is true for the others.

Among the top 100 optimal crosses out of the 64,980 ones for 100SW, 13 crosses had both parents from the top 10 100SW accessions, and 52 crosses had only one parent from the top 10 100SW accessions (**Supplementary Table 5**; **Figure 2G**). There were 35 crosses that did not include any of the top 10 100SW accession. Similarly, among the top 100 optimal crosses for SOC, there were 14 crosses with both parents from the top 10 SOC accessions, and 48 crosses with only one parent from the top 10 SOC accessions (**Supplementary Table 6**; **Figure 2H**). Additionally, 38 crosses did not involve any of the top 10 SOC accessions. Even some were involved with the top ones, but not ranking in the front position. This indicated that the top 10 accessions might play an important role in breeding for 100SW and SOC, but a breakthrough improvement may not be readily achieved through crossing the best accessions only. On the other hand, the best predicted cross for SOC had its parent without any of the top 10 SOC accessions. This indicated that complementary potential lay in specific pairs of the accessions, not necessarily in only the best accessions of the NECSGP. Therefore, evaluation of each accession’s QTL-allele constitution for finding the best complementary potential pairs is necessary in a germplasm/breeding population. In other words, for 100SW and SOC in the NECSGP, “specific combining ability” or “specific recombination potential” is more important.

Although there were significant differences in 100SW and SOC among different maturity groups, the predicted recombination potentials within MG I+II+III, MG 0 + 00 + 000, and MG 0 differed slightly from others with the 95th maximum percentile of 100SW being more than 28.57 g and that of SOC being more than 27.12%. Those between MG 0 + 00 + 000 and MG I+II+III, MG 0 and MG I+II+III, MG 00 and MG I+II+III, and MG 000 and MG I+II+III showed more recombination potential than the others with the 95th maximum percentile of 100SW being more than 29.99 g and that of SOC being more than 27.73%. It means that crosses from different MGs, especially those crossed with the late MGs (MG I+II+III), might have more potential in 100SW and SOC due to a broader genetic background while the crosses between the early maturity groups have less potential due to a narrowed genetic background (**Table 4**).

To design optimal crosses for the simultaneous improvement of 100SW and SOC, three strategies were applied. Firstly, sequential selection was applied to screen optimal crosses for simultaneous improvement in 100SW and SOC. Taking 100SW as the priority, the top 10 crosses with the highest SOC were selected from the 100 optimal crosses for 100SW, and marked as 100SW-first crosses for simultaneous improvement in 100SW and SOC (**Table 5**). The recombination potential of the top 10 100SW-first crosses ranged from 27.96 to 30.33 g for 100SW while that of SOC ranged from 24.79% to 25.82%. On the other hand, taking SOC as the priority, the top 10 crosses with the highest 100SW were selected from the 100 optimal crosses for SOC, and marked as the SOC-first crosses for simultaneous improvement in 100SW and SOC. The recombination potential of the top 10 SOC-first crosses ranged from 26.60% to 27.11% while that of 100SW ranged from 22.55 to 24.07 g. As for the 100SW-SOC-balance strategy, the top 10 joint crosses were selected by lining up all the predicted crosses in descending order for 100SW and SOC, respectively. The top 10 common crosses from both sides were marked as the 100SW-SOC-balance crosses for simultaneous improvement of 100SW and SOC. Its predicted recombination potential ranged from 25.48 to 26.29 g for 100SW and from 25.95% to 26.12% for SOC.

**Table 5:** Optimal crosses selected for simultaneous improvement of 100SW and SOC in the NECSGP.

Strategy	Order	P1				P2				Predicted progeny	
		ID	MG	100SW	SOC	ID	MG	100SW	SOC	100SW	SOC
100SW-first	1	***F32***	0	20.94	24.31	F35	III	20.78	20.98	28.11	25.82
	2	**F406**	0	24.15	22.60	P087	I	21.84	23.70	28.35	25.28
	3	F229	0	20.54	21.95	F364	0	19.89	22.98	28.33	25.00
	4	F35	III	20.78	20.98	F49	0	20.45	23.06	28.09	24.98
	5	**F326**	III	24.28	20.38	P087	I	21.84	23.70	28.23	24.98
	6	**F58**	0	23.84	22.60	P087	I	21.84	23.70	27.97	24.86
	7	F28	0	21.56	23.41	F35	III	20.78	20.98	27.96	24.85
	8	F49	0	20.45	23.06	**F67**	III	25.16	21.00	28.19	24.82
	9	F312	0	22.78	23.06	F35	III	20.78	20.98	28.64	24.80
	10	**F326**	III	24.28	20.38	F364	0	19.89	22.98	30.33	24.79
		**Mean**								28.42	25.02
SOC-first	1	***F386***	0	19.34	24.54	F49	0	20.45	23.06	24.07	26.80
	2	***F386***	0	19.34	24.54	***F79***	0	21.04	24.33	23.77	26.77
	3	***F32***	0	20.94	24.31	***F386***	0	19.34	24.54	23.48	26.91
	4	***F32***	0	20.94	24.31	F343	II	18.84	23.45	23.46	27.11
	5	***F305***	0	17.46	24.16	***F32***	0	20.94	24.31	23.24	26.60
	6	***F306***	0	17.83	24.10	***F32***	0	20.94	24.31	23.14	26.69
	7	F140	0	18.29	23.88	***F79***	0	21.04	24.33	22.91	26.65
	8	F109	II	17.29	24.00	F313	0	22.18	23.05	22.84	26.62
	9	F244	0	20.21	23.70	F343	II	18.84	23.45	22.73	26.71
	10	F343	II	18.84	23.45	***F386***	0	19.34	24.54	22.55	26.93
		**Mean**								23.22	26.78
100SW-SOC-balance	1	F309	0	22.66	23.33	***F386***	0	19.34	24.54	25.51	26.12
	2	F197	0	20.05	22.51	***F32***	0	20.94	24.31	25.48	26.09
	3	***F53***	0	20.58	24.19	F087	I	21.84	23.7	25.54	26.07
	4	F109	II	17.29	24.00	F35	III	20.78	20.98	25.45	26.07
	5	F140	0	18.29	23.88	F309	0	22.66	23.33	25.70	26.05
	6	F344	II	16.29	24.01	F364	0	19.89	22.98	25.63	25.99
	7	F109	II	17.29	24.00	F364	0	19.89	22.98	25.49	25.96
	8	***F32***	0	20.94	24.31	P087	I	21.84	23.70	26.29	25.95
	9	***F306***	0	17.83	24.10	P087	I	21.84	23.70	25.54	25.95
	10	F140	0	18.29	23.88	F35	III	20.78	20.98	25.96	25.95
		**Mean**								25.66	26.02

P1, P2, parents 1 and 2 of a cross; ID, accession name; MG, maturity group; 100SW, 100-seed weight (g); SOC, seed oil content (%); 100SW- and SOC-first, the top 10 optimal crosses selected from the 95% percentile of offspring of 100SW and SOC. 100SW-SOC-balance, the predicted respective 100SW and SOC crosses were arranged in descending order, and the top 10 joint crosses from both sides were selected as the best crosses for balanced 100SW and SOC. The parental accessions in boldface are the top 10 100SW accessions, i;e., F67, F326, F406, F147, F58, P004, P085, F36, P188, and F315 in descending order, and those in italic boldface are the top 10 SOC accessions, i;e;, F82, F386, F135, F79, F32, F155, F53, F305, F306, and F351 in descending order in the NECSGP.

## 4 Discussion

### 4.1 Relative completeness and comparability in dissecting the QTL-allele systems of 100SW and SOC in the NECSGP through RTM-GWAS

A thorough detection of all QTLs and their multiple alleles is the key to efficient utilization of superior genes–alleles in the germplasm population in plant breeding. In this study, the QTL-allele system of 100SW and SOC in the NECSGP was detected using the RTM-GWAS procedure. A large amount of the genetic variation of these two seed traits was explained by the detected QTLs, both large- and small-contribution QTLs, as well as their alleles. Many QTLs had a phenotype contribution of less than *R* ^2^ < 1%, but altogether explained 19.51% and 16.96% phenotypic variation for 100SW and SOC in the NECSGP, respectively. Despite the high efficiency of RTM-GWAS in whole-genome QTL-allele detection, there were still unmapped genetic variations (28.27% and 16.54% for 100SW and SOC, respectively) that remained to be explored through further improvement of experiment resolution. The QTLs with a significant interaction effect with the environment were also detected in this study, but the phenotypic variation explained by the QTL × environment interaction (QEI) was relatively smaller than the main effect QTL. This may be due to the variability of the random environment factor (different years) in this study.

For comparison of QTLs detected in this study with previous studies, the worldwide information of 310 and 325 QTLs of 100SW and SOC at SoyBase (https://www.soybase.org) was retrieved, respectively. A total of 106 and 131 QTLs with a supporting interval of less than two centimorgans were used for comparisons with 23 and 32 QTLs overlapping to previously reported 100-SW and SOC QTLs, respectively. Previous results were mainly obtained from bi-parental populations using linkage mapping under different environments, whereas the present study utilized the NECSGP in association mapping under a uniform environment. Consequently, the identified QTL-allele system of 100SW and SOC should be relatively more comprehensive and comparable in NEC. This may be more relevant to the genetic operation of the QTL-allele systems in breeding for cultivars fitting the requirements under the environmental conditions of NEC, to design the optimal crosses in the present study.

From the above, the relative completeness and comparability in dissecting the QTL-allele systems of 100SW and SOC in the NECSGP are mainly through RTM-GWAS. This is due to the large germplasm population with broad genetic variation and the increased efficiency with two innovative procedures as indicated in the Introduction section.

### 4.2 Genome-wide QTL-allele dissection of a germplasm or breeding population helps the optimal utilization of complementary alleles

Recombination breeding is based on the QTL-allele or gene-allele dissection of the breeding or germplasm population, for which the establishment of a QTL-allele matrix through RTM-GWAS provided a promising approach to realize the breeders’ targets. Previously, the breeders usually design their crosses based on the phenotypic values of their parental lines. Now, based on the identification of the genome-wide QTL-allele or gene-allele system, the breeders can design crosses genetically. However, sometimes the geneticists consider only individual genes with their alleles while the breeders have to consider the whole QTL-allele or gene-allele system because they do not want to leave inferior ones in a cultivar’s genetic background, especially for complex traits.

In **Table 5**, **Supplementary Tables 5** and **6**, the optimal crosses for single traits and two simultaneous traits are selected with the top 10 parents for each of the two traits marked. Not many predicted crosses with their two parents located in the top 10 accessions for each trait in the NECSGP; only a portion of predicted crosses have one parent located in the top 10 accessions for each trait in the NECSGP; even many predicted crosses do not have their one parent from the top 10 accessions for each trait in the NECSGP. This means that the complementary alleles scattered in different parental accessions. This further emphasizes the importance of whole-genome dissection for the utilization of the whole-genome complementary potential.

In addition, in the evolution from late to early maturity groups, allele emergence was quite limited whereas allele exclusion was fluctuating; this fact also supports that the evolutionary changes may be caused by the release of complementary potential due to many recombinations in history that generate phenotypic variations among maturity groups.

### 4.3 Genomic selection for multi-trait optimal cross design

The breeding by design concept was proposed for designing optimal crosses and selecting superior progeny, in which detecting the QTLs and their alleles was its prerequisite (Peleman and van der Voort, 2003). Breeding by design provided a basic framework for pyramiding a few major genes. Since a quantitative trait is generally controlled by numerous QTLs or genes, plant breeding breakthrough should involve in fact most QTLs and their alleles, including both large and small effect loci. Therefore, the germplasm population rather than the bi-parental population should be studied for a relatively thorough detection of the QTL-allele system. In this study, computer simulation was used to generate the progeny genotype, and the progeny genotypic value was predicted using the QTL-allele matrix. Then, optimal crosses can be selected based on progeny phenotype distribution (e.g., the 95th percentile). This *in silico* work is efficient and could not be done in the field because of the impossible large scale. The QEI QTL-allele data set can also be organized into a matrix if it is needed (Fu et al., 2020b). However, the environmental factor in the present study varied randomly, and no fixed environmental parameter was available to provide useful information in breeding for quantitative traits. Therefore, the QEI information was not used in the present analysis.

In this study, 100SW and SOC were simultaneously considered for optimal cross design. Since tight linkage between loci may exist, the two traits cannot be improved independently to achieve an ideal situation. To address this challenge, this study proposed three types of optimal crosses for the simultaneous improvement of 100SW and SOC in the NECSGP. The 100SW-first optimal crosses prioritized improving 100SW as the primary focus, with SOC being considered as the secondary objective. In contrast, the SOC-first optimal crosses focused on SOC at first and then 100SW. In addition, 100SW-SOC-balance optimal crosses focused not on the extreme value of one trait, but the best overall. Instead of separately focusing on the extreme values of one trait, this study aimed to also identify optimal crosses that would result in the best overall improvement of both 100SW and SOC. Here, the top 2,000 crosses were used for ordering in each trait. It depends on the number cases, and 2,000 crosses for each trait are enough in this study. Based on these three types of optimal crosses, this study presented a preliminary attempt at multi-trait cross design in the NECSGP. The improvement of 100SW and SOC responding to the three simultaneous selection strategies may raise both traits’ levels, but different from each other. The researchers can choose their preferred one based on their requirements. For example, the SOC-first strategy may be considered because SOC is economically more important than 100SW. In addition, the method can be extended to simultaneous selection for more traits. However, it is important to note that the methods based on a composite selection index may offer more sophisticated approaches for multi-trait selection in breeding.

The concept of GS using genome-wide markers was introduced as a method of predicting breeding values of a group of traits. This approach, proposed by Meuwissen et al. (2001), aimed to leverage the information provided by the entire genome rather than focusing on specific loci of some specific traits, especially in animal breeding. In classical GS, it is assumed that all markers across the genome have effects on the trait of interest. These effects are then estimated using a reference or training population, which serves as the basis for establishing a prediction model. By considering the collective influence of multiple markers, GS offers the potential to capture the polygenic nature of many complex traits. However, it is important to note that genetic mechanisms governing different quantitative traits can vary significantly. The complexity of these mechanisms can also differ, making it challenging to apply a uniform approach to all traits. Therefore, it becomes crucial to consider the specific characteristics of the trait under consideration when implementing GS.

The use of QTL-allele matrices allows for a more targeted and tailored approach to GS. By focusing on the specific alleles associated with QTLs, breeders can gain insights into the genetic basis of the trait and make informed decisions regarding optimal crosses. This approach acknowledges the unique characteristics and complexities of different quantitative traits, offering a more suitable framework for designing breeding strategies in plants. Therefore, genome-wide breeding by design in fact is the GS based on the genome-wide QTL-allele (or gene-allele) matrix, which should be more appropriate in designing optimal crosses in plants.

## Data Availability

The data presented in the study are deposited in the GitHub repository, accession number: https://github.com/njau-sri/NECSGP-100SW-SOC.
