# Supplementary material for: Identification of QTL-allele systems of seed size and oil content for simultaneous genomic improvement in Northeast China soybeans
Source: Front Plant Sci. 2024 Nov 14;15:1483995. doi: 10.3389/fpls.2024.1483995 (PMC11602309; doi:10.3389/fpls.2024.1483995)
Supplement: Supplementary file 1 [file Table1.docx]

**Table S1 Joint analysis of variance of 100-seed weight and seed oil content in the NECSGP**

| Source | 100-seed weight | | | | seed oil content | | | |
| --- | --- | --- | --- | --- | --- | --- | --- | --- |
|  | DF | SS | MS | F | DF | SS | MS | F |
| Year. | 1 | 2498.43 | 2498.43 | 82.69** | 1 | 11.96 | 11.96 | 7.13* |
| Block (Year) | 6 | 163.76 | 27.29 | 7.6** | 6 | 6.27 | 1.05 | 3.54** |
| Genotype | 360 | 13364 | 37.12 | 5.68** | 360 | 2445.17 | 6.79 | 7.28** |
| Genotype×Year | 360 | 2354.19 | 6.54 | 1.82** | 356 | 332.23 | 0.93 | 3.16** |
| Error | 2141 | 7691.31 | 3.59 |  | 2129 | 629.33 | 0.30 |  |
| Total | 2868 | 26091.00 |  |  | 2852 | 3413.19 |  |  |

*, significant difference at a level of 0.05; **, significant difference at a level of 0.01.

**Table S2 The distribution and descriptive statistics of 100-seed weight and seed oil content in different maturity groups of the NECSGP**

| Trait | MG | Mid-point | | | | | | | | | | | | | *N* | Mean | Range |
| --- | --- | --- | --- | --- | --- | --- | --- | --- | --- | --- | --- | --- | --- | --- | --- | --- | --- |
| 100SW |  | 9.0 | 10.5 | 12.0 | 13.5 | 15.0 | 16.5 | 18.0 | 19.5 | 21.0 | 22.5 | 24.0 | 25.5 | 27.0 |  |  |  |
|  | 000 |  |  |  | 1 | 0 | 2 | 6 | 4 | 1 | 4 | 2 | 1 |  | 16 | 19.25^ab^ | 13.97-25.16 |
|  | 00 |  | 1 | 0 | 0 | 1 | 4 | 10 | 16 | 7 | 6 |  |  |  | 45 | 19.24^ab^ | 10.99-22.78 |
|  | 0 |  |  |  | 3 | 6 | 38 | 41 | 38 | 22 | 5 | 4 |  |  | 157 | 18.54^bc^ | 13.20-24.15 |
|  | Ⅰ |  |  |  | 1 | 9 | 24 | 21 | 19 | 4 | 1 |  |  |  | 79 | 17.70^cd^ | 13.75-21.84 |
|  | Ⅱ | 1 | 0 | 0 | 0 | 6 | 17 | 14 | 3 | 1 | 1 |  |  |  | 43 | 17.03^d^ | 9.09-23.05 |
|  | Ⅲ |  |  |  | 1 | 0 | 2 | 6 | 4 | 1 | 4 | 2 | 1 |  | 21 | 19.72^a^ | 13.97-25.16 |
| SOC |  | 19.0 | 19.5 | 20.0 | 20.5 | 21.0 | 21.5 | 22.0 | 22.5 | 23.0 | 23.5 | 24.0 | 24.5 | 25.0 |  |  |  |
|  | 000 |  |  |  | 1 | 0 | 0 | 7 | 3 | 2 | 2 | 1 |  |  | 16 | 22.45^b^ | 20.6-23.79 |
|  | 00 |  |  |  |  |  | 5 | 6 | 5 | 10 | 17 | 2 |  |  | 45 | 22.89^a^ | 21.26-24.05 |
|  | 0 |  |  |  | 2 | 4 | 9 | 28 | 40 | 33 | 28 | 7 | 6 |  | 157 | 22.71^ab^ | 20.41-24.73 |
|  | Ⅰ |  |  |  |  | 2 | 11 | 18 | 22 | 15 | 10 | 1 |  |  | 79 | 22.47^b^ | 21.14-23.79 |
|  | Ⅱ |  |  | 1 | 8 | 6 | 7 | 7 | 8 | 2 | 2 | 2 |  |  | 43 | 21.68^c^ | 19.9-24.01 |
|  | Ⅲ | 1 | 0 | 2 | 4 | 4 | 5 | 5 |  |  |  |  |  |  | 21 | 21.07^d^ | 19.21-22.14 |

MG, maturity group with the latest III and the earliest 000. 100SW, 100-seed weight (g); SOC, seed oil content (%). *N*, the number of accessions. Letters in lowercase (a,b,c,d) at the top right corner of mean values indicate significance test for phenotype differences among maturity groups.

**Table S3 The QTLs/SNPLDBs associated with 100-seed weight in the NECSGP**

|  |  |  | Model | QTL main | | QTLxYear | |
| --- | --- | --- | --- | --- | --- | --- | --- |
| QTL | Position (bp) | AN | -lg*P* | -lg*P* | *R*^2^(%) | -lg*P* | *R*^2^(%) |
| *qSW-1-1* | 17738048 | 2 | 1.7 |  |  | 1.7 | 0.09 |
| *qSW-1-2* | 18083704 | 2 | 2.0 | 1.8 | 0.09 |  |  |
| *qSW-1-3* | 39719863-39752334 | 3 | 3.5 | 2.2 | 0.16 | 3.2 | 0.23 |
| *qSW-1-4* | 45469815 | 2 | 17.4 | 35.7 | 2.61 |  |  |
| *qSW-2-1* | 5834794 | 2 | 3.0 |  |  | 3.5 | 0.21 |
| *qSW-2-2* | 13721101-13737254 | 3 | 4.2 | 5.9 | 0.43 |  |  |
| *qSW-3-1* | 15467787 | 2 | 1.4 | 1.8 | 0.10 |  |  |
| *qSW-3-2* | 16035237-16035286 | 2 | 3.0 | 3.1 | 0.18 |  |  |
| *qSW-4-1* | 1589459-1605811 | 3 | 6.0 | 8.9 | 0.66 |  |  |
| *qSW-4-2* | 3959848-3999358 | 5 | 13.9 | 31.1 | 2.50 |  |  |
| *qSW-4-3* | 9622583-9649077 | 4 | 3.0 | 3.8 | 0.33 |  |  |
| *qSW-4-4* | 9903141 | 2 | 3.3 |  |  | 3.7 | 0.22 |
| *qSW-4-5* | 26561037 | 2 | 1.4 |  |  | 1.8 | 0.09 |
| *qSW-5-1* | 2558192 | 2 | 1.5 | 2.6 | 0.14 |  |  |
| *qSW-5-2* | 27603689-27803518 | 3 | 8.2 | 11.9 | 0.88 |  |  |
| *qSW-5-3* | 32544883 | 2 | 4.9 | 6.8 | 0.44 |  |  |
| *qSW-5-4* | 33761407 | 2 | 10.0 | 17.6 | 1.23 |  |  |
| *qSW-5-5* | 35789875 | 2 | 5.5 | 5.3 | 0.34 | 3.0 | 0.17 |
| *qSW-6-1* | 1086623 | 2 | 4.8 | 2.6 | 0.15 | 4.6 | 0.28 |
| *qSW-6-2* | 15569110-15713936 | 6 | 11.3 | 21.8 | 1.82 |  |  |
| *qSW-6-3* | 31084754-31225436 | 6 | 4.1 | 5.4 | 0.52 |  |  |
| *qSW-6-4* | 32166254-32180510 | 5 | 12.2 | 23.1 | 1.87 | 1.7 | 0.18 |
| *qSW-7-1* | 1936828-1953342 | 3 | 6.0 | 8.5 | 0.63 |  |  |
| *qSW-7-2* | 10873765-10928978 | 4 | 2.2 | 2.1 | 0.19 |  |  |
| *qSW-7-3* | 12335347 | 2 | 3.7 | 4.5 | 0.28 |  |  |
| *qSW-7-4* | 36692391-36692601 | 2 | 2.7 | 3.4 | 0.20 |  |  |
| *qSW-8-1* | 25919688 | 2 | 2.8 | 1.8 | 0.09 | 2.5 | 0.14 |
| *qSW-8-2* | 29842887 | 2 | 18.8 | 39.6 | 2.92 |  |  |
| *qSW-8-3* | 30320715-30453625 | 8 | 4.3 | 3.8 | 0.46 | 3.2 | 0.41 |
| *qSW-8-4* | 36491999-36691991 | 2 | 3.0 | 2.7 | 0.15 | 1.6 | 0.08 |
| *qSW-8-5* | 37454764 | 2 | 12.1 | 23.5 | 1.68 |  |  |
| *qSW-9-1* | 5426214-5440654 | 3 | 9.4 | 12.2 | 0.91 | 4.4 | 0.33 |
| *qSW-9-2* | 13502134 | 2 | 1.9 | 2.8 | 0.16 |  |  |
| *qSW-9-3* | 16774220 | 2 | 9.1 | 15.1 | 1.05 |  |  |
| *qSW-9-4* | 17858774 | 2 | 4.1 | 5.6 | 0.35 |  |  |
| *qSW-9-5* | 31770727 | 2 | 3.0 | 1.3 | 0.06 | 3.1 | 0.18 |
| *qSW-10-1* | 2911784-2972949 | 5 | 9.5 | 16.8 | 1.38 | 2.4 | 0.25 |
| *qSW-10-2* | 4360003 | 2 | 3.2 | 4.2 | 0.25 |  |  |
| *qSW-10-3* | 25452676 | 2 | 1.5 | 2.9 | 0.17 |  |  |
| *qSW-10-4* | 37955786-37958277 | 2 | 2.6 | 2.0 | 0.11 | 1.7 | 0.09 |
| *qSW-10-5* | 45609679 | 2 | 2.7 |  |  | 3.0 | 0.17 |
| *qSW-10-6* | 48538275 | 2 | 12.6 | 23.0 | 1.64 |  |  |
| *qSW-11-1* | 1070812 | 2 | 3.4 | 3.0 | 0.17 | 2.1 | 0.11 |
| *qSW-11-2* | 8567759-8763159 | 5 | 36.3 | 80.7 | 6.58 | 1.8 | 0.19 |
| *qSW-11-3* | 32989073-33006432 | 2 | 2.5 | 1.8 | 0.09 | 1.9 | 0.10 |
| *qSW-12-1* | 2385662 | 2 | 4.3 | 5.8 | 0.37 |  |  |
| *qSW-12-2* | 4943597 | 2 | 3.1 | 2.3 | 0.13 | 2.3 | 0.12 |
| *qSW-12-3* | 17580420-17777027 | 4 | 7.8 | 11.2 | 0.89 | 2.2 | 0.20 |
| *qSW-12-4* | 26279858-26279925 | 2 | 3.1 | 4.0 | 0.24 |  |  |
| *qSW-12-5* | 28338869 | 2 | 3.3 | 3.4 | 0.20 | 1.6 | 0.08 |
| *qSW-13-1* | 4913400 | 2 | 1.5 | 2.2 | 0.12 |  |  |
| *qSW-13-2* | 15686789-15686799 | 2 | 5.4 | 3.9 | 0.23 | 4.5 | 0.27 |
| *qSW-13-3* | 16403751 | 2 | 5.8 | 7.2 | 0.47 | 2.0 | 0.10 |
| *qSW-13-4* | 26799133-26841802 | 3 | 8.0 | 12.8 | 0.95 |  |  |
| *qSW-13-5* | 37385012 | 2 | 6.2 | 5.8 | 0.37 | 3.8 | 0.23 |
| *qSW-13-6* | 38015705 | 2 | 6.2 | 7.9 | 0.52 | 1.7 | 0.09 |
| *qSW-13-7* | 40173283-40173381 | 2 | 4.7 | 3.6 | 0.21 | 3.6 | 0.21 |
| *qSW-14-1* | 2906061 | 2 | 1.6 | 2.3 | 0.13 |  |  |
| *qSW-14-2* | 17268836 | 2 | 3.8 | 5.0 | 0.31 |  |  |
| *qSW-14-3* | 44039856 | 2 | 5.9 | 8.3 | 0.55 |  |  |
| *qSW-14-4* | 46936197 | 2 | 8.3 | 13.6 | 0.94 |  |  |
| *qSW-15-1* | 27245942 | 2 | 2.1 | 1.5 | 0.07 |  |  |
| *qSW-15-2* | 41068848 | 2 | 7.3 | 10.8 | 0.73 |  |  |
| *qSW-17-1* | 1426143-1454753 | 4 | 9.5 | 14.8 | 1.17 | 2.0 | 0.18 |
| *qSW-17-2* | 2124262 | 2 | 2.2 |  |  | 2.1 | 0.11 |
| *qSW-17-3* | 3962122 | 2 | 2.8 | 2.4 | 0.13 | 1.9 | 0.10 |
| *qSW-17-4* | 24164909 | 2 | 1.8 |  |  | 2.4 | 0.13 |
| *qSW-17-5* | 26654610-26854593 | 7 | 13.9 | 32.2 | 2.71 |  |  |
| *qSW-17-6* | 35769952-35842671 | 6 | 6.3 | 8.8 | 0.80 | 2.5 | 0.29 |
| *qSW-18-1* | 4558941 | 2 | 1.9 | 3.1 | 0.18 |  |  |
| *qSW-19-1* | 2239196 | 2 | 3.7 |  |  | 4.9 | 0.31 |
| *qSW-19-2* | 12013875-12212796 | 8 | 6.2 | 5.6 | 0.62 | 4.5 | 0.53 |
| *qSW-19-3* | 18155106 | 2 | 8.0 | 12.2 | 0.83 |  |  |
| *qSW-19-4* | 23816470-23847882 | 7 | 12.0 | 25.5 | 2.17 | 2.2 | 0.29 |
| *qSW-19-5* | 24429545-24450383 | 5 | 2.5 | 2.6 | 0.19 |  |  |
| *qSW-19-6* | 48261855-48305156 | 4 | 14.4 | 27.7 | 2.16 | 1.6 | 0.15 |
| *qSW-20-1* | 15194157 | 2 | 1.4 | 2.3 | 0.13 |  |  |
| *qSW-20-2* | 26034448-26053854 | 3 | 8.7 | 14.9 | 1.11 |  |  |
| *qSW-20-3* | 37939436-38127044 | 3 | 2.9 | 1.7 | 0.13 | 2.5 | 0.18 |
| *qSW-20-4* | 43520063-43697026 | 6 | 3.8 | 3.6 | 0.38 | 2.1 | 0.25 |
| **Total** |  | **232** | **80** | **72** | **54.11** | **38** | **7.34** |

A QTL, such as *qSW-1-2*, is designated in the following way: "SW" means 100-seed weight, "-1" represents chromosome 1, and "-2" represents its order (the second) on the chromosome according to the position.

AN, number of alleles.

**Table S4 The QTLs/SNPLDBs associated with seed oil content in the NECSGP**

|  |  |  | Model | QTL main |  | QTLxYear |  |
| --- | --- | --- | --- | --- | --- | --- | --- |
| QTL | Position (bp) | AN | -lg*P* | -lg*P* | *R*^2^(%) | -lg*P* | *R*^2^(%) |
| *qSOC-1-1* | 53359897 | 2 | 7.4 |  |  | 11.0 | 0.42 |
| *qSOC-2-1* | 12540482 | 2 | 1.5 |  |  | 2.0 | 0.06 |
| *qSOC-2-2* | 15613873 | 2 | 1.3 | 1.5 | 0.04 |  |  |
| *qSOC-2-3* | 19518514 | 2 | 16.5 | 43.3 | 1.80 | 2.9 | 0.09 |
| *qSOC-2-4* | 23178890-23211938 | 4 | 10.6 | 20.7 | 0.91 |  |  |
| *qSOC-2-5* | 23960254 | 2 | 6.3 | 9.3 | 0.35 |  |  |
| *qSOC-2-6* | 33127066-33140987 | 2 | 1.6 |  |  | 1.5 | 0.04 |
| *qSOC-2-7* | 36735281-36934976 | 8 | 6.0 | 7.8 | 0.45 | 2.0 | 0.16 |
| *qSOC-2-8* | 48382122-48391378 | 2 | 3.7 | 4.7 | 0.16 |  |  |
| *qSOC-2-9* | 50652540 | 2 | 1.4 | 1.6 | 0.04 |  |  |
| *qSOC-3-1* | 14699738-14752188 | 6 | 2.7 | 3.8 | 0.22 |  |  |
| *qSOC-3-2* | 18922646-19094931 | 3 | 8.8 | 12.2 | 0.51 |  |  |
| *qSOC-3-3* | 40883791-40983710 | 7 | 18.0 | 57.8 | 2.69 | 8.0 | 0.44 |
| *qSOC-4-1* | 7173325-7349829 | 6 | 7.0 | 7.5 | 0.39 | 3.6 | 0.21 |
| *qSOC-4-2* | 22829878 | 2 | 9.0 | 12.6 | 0.48 | 2.0 | 0.06 |
| *qSOC-4-3* | 23851723 | 2 | 9.5 | 15.4 | 0.60 | 2.0 | 0.06 |
| *qSOC-4-4* | 27993724 | 2 | 14.0 | 34.4 | 1.41 |  |  |
| *qSOC-4-5* | 43821525-43868385 | 5 | 5.4 | 1.9 | 0.11 | 6.4 | 0.32 |
| *qSOC-5-1* | 1282 | 2 | 1.6 |  |  | 2.0 | 0.06 |
| *qSOC-5-2* | 15751165 | 2 | 1.4 | 1.7 | 0.05 |  |  |
| *qSOC-6-1* | 4187478-4265060 | 4 | 15.1 | 39.5 | 1.74 | 3.5 | 0.17 |
| *qSOC-6-2* | 25148440 | 2 | 11.6 | 18.3 | 0.72 | 1.4 | 0.04 |
| *qSOC-6-3* | 37273090-37273106 | 3 | 15.3 | 33.5 | 1.42 |  |  |
| *qSOC-6-4* | 40233618 | 2 | 4.2 | 4.9 | 0.17 |  |  |
| *qSOC-6-5* | 45623643 | 2 | 19.3 | 48.1 | 2.01 | 7.0 | 0.26 |
| *qSOC-6-6* | 49599694-49713025 | 6 | 10.3 | 22.4 | 1.05 |  |  |
| *qSOC-7-1* | 3765316 | 2 | 1.5 |  |  | 2.1 | 0.06 |
| *qSOC-7-2* | 8219018 | 2 | 1.6 |  |  | 2.1 | 0.06 |
| *qSOC-7-3* | 15404334 | 2 | 1.4 |  |  | 1.3 | 0.03 |
| *qSOC-7-4* | 22183028-22273636 | 5 | 13.8 | 35.4 | 1.59 | 2.8 | 0.16 |
| *qSOC-7-5* | 33303110 | 2 | 3.6 | 4.8 | 0.17 |  |  |
| *qSOC-7-6* | 36933382-36940967 | 5 | 11.8 | 18.4 | 0.84 | 3.8 | 0.20 |
| *qSOC-7-7* | 41357866 | 2 | 14.5 | 32.1 | 1.31 |  |  |
| *qSOC-8-1* | 25666752 | 2 | 2.7 | 2.3 | 0.07 | 1.7 | 0.05 |
| *qSOC-8-2* | 35664728 | 2 | 6.4 | 7.2 | 0.26 | 3.3 | 0.11 |
| *qSOC-9-1* | 8389899-8413454 | 2 | 2.0 | 2.0 | 0.06 |  |  |
| *qSOC-9-2* | 11717949 | 2 | 2.6 | 2.3 | 0.07 | 1.5 | 0.04 |
| *qSOC-9-3* | 21619226-21621904 | 2 | 10.4 | 19.0 | 0.75 | 2.1 | 0.06 |
| *qSOC-9-4* | 24292065 | 2 | 10.3 | 13.7 | 0.53 | 4.8 | 0.17 |
| *qSOC-9-5* | 24463145 | 2 | 2.3 | 2.4 | 0.07 |  |  |
| *qSOC-9-6* | 34615681-34744576 | 6 | 2.7 | 1.9 | 0.13 | 2.0 | 0.14 |
| *qSOC-9-7* | 36738268-36738288 | 2 | 12.3 | 24.3 | 0.97 |  |  |
| *qSOC-9-8* | 39523118-39600466 | 6 | 23.5 | 87.4 | 4.07 | 1.7 | 0.12 |
| *qSOC-9-9* | 39718665 | 2 | 3.7 | 2.1 | 0.06 | 3.1 | 0.10 |
| *qSOC-9-10* | 43399724-43570001 | 5 | 14.1 | 34.0 | 1.53 |  |  |
| *qSOC-10-1* | 6377182-6451543 | 5 | 13.9 | 33.1 | 1.49 |  |  |
| *qSOC-10-2* | 9953697-10151797 | 5 | 11.2 | 22.9 | 1.04 | 3.5 | 0.19 |
| *qSOC-10-3* | 26439102-26634629 | 7 | 13.8 | 24.2 | 1.16 | 14.9 | 0.75 |
| *qSOC-10-4* | 32546004 | 2 | 4.6 | 5.7 | 0.20 |  |  |
| *qSOC-10-5* | 46728553-46730774 | 2 | 3.5 | 3.0 | 0.10 | 2.0 | 0.06 |
| *qSOC-11-1* | 7602695-7800774 | 6 | 22.4 | 83.2 | 3.87 | 6.5 | 0.34 |
| *qSOC-11-2* | 37289931-37466750 | 3 | 1.8 | 1.8 | 0.07 |  |  |
| *qSOC-12-1* | 10419180-10443940 | 4 | 20.5 | 64.1 | 2.86 |  |  |
| *qSOC-12-2* | 11412635 | 2 | 2.2 | 2.8 | 0.09 |  |  |
| *qSOC-12-3* | 18481215 | 2 | 6.2 | 2.0 | 0.06 | 7.5 | 0.28 |
| *qSOC-12-4* | 29795721 | 2 | 14.2 | 32.1 | 1.31 |  |  |
| *qSOC-12-5* | 32995809-33159018 | 4 | 8.3 | 13.8 | 0.61 |  |  |
| *qSOC-13-1* | 7017154-7038684 | 5 | 2.4 | 1.7 | 0.10 | 1.8 | 0.11 |
| *qSOC-13-2* | 9922025-10011933 | 6 | 17.2 | 59.5 | 2.74 | 4.4 | 0.25 |
| *qSOC-13-3* | 14479592 | 2 | 4.7 |  |  | 5.7 | 0.20 |
| *qSOC-13-4* | 18248501 | 2 | 5.4 | 7.4 | 0.27 |  |  |
| *qSOC-13-5* | 18570867 | 2 | 10.9 | 17.2 | 0.68 |  |  |
| *qSOC-13-6* | 25308006-25504934 | 5 | 4.5 | 5.7 | 0.29 |  |  |
| *qSOC-13-7* | 36993297 | 2 | 11.0 | 21.0 | 0.83 |  |  |
| *qSOC-14-1* | 14662041 | 2 | 3.5 | 4.5 | 0.16 |  |  |
| *qSOC-14-2* | 17268836 | 2 | 3.3 | 4.3 | 0.15 |  |  |
| *qSOC-14-3* | 21409536 | 2 | 6.3 | 2.6 | 0.08 | 7.0 | 0.26 |
| *qSOC-14-4* | 27778940 | 2 | 15.6 | 41.8 | 1.73 |  |  |
| *qSOC-15-1* | 12711686-12899256 | 6 | 9.8 | 18.4 | 0.87 |  |  |
| *qSOC-16-1* | 18526618 | 2 | 2.2 |  |  | 2.5 | 0.08 |
| *qSOC-17-1* | 4885563-5078835 | 7 | 22.1 | 77.9 | 3.66 | 5.7 | 0.33 |
| *qSOC-17-2* | 12316166-12450640 | 5 | 10.4 | 17.8 | 0.81 | 3.3 | 0.18 |
| *qSOC-17-3* | 17785324 | 2 | 2.5 | 2.0 | 0.06 | 1.7 | 0.05 |
| *qSOC-17-4* | 33012195 | 2 | 6.7 | 2.6 | 0.08 | 8.2 | 0.30 |
| *qSOC-18-1* | 525897-570837 | 3 | 22.3 | 83.5 | 3.72 |  |  |
| *qSOC-18-2* | 2334449-2348078 | 5 | 25.5 | 101.0 | 4.70 | 3.5 | 0.19 |
| *qSOC-18-3* | 7925273-7949344 | 4 | 4.4 | 4.3 | 0.20 | 2.0 | 0.10 |
| *qSOC-18-4* | 26720348 | 2 | 3.3 | 4.2 | 0.15 |  |  |
| *qSOC-18-5* | 31721367 | 2 | 5.0 | 2.5 | 0.08 | 4.8 | 0.17 |
| *qSOC-18-6* | 37130298 | 2 | 2.8 |  |  | 3.6 | 0.12 |
| *qSOC-18-7* | 40742222 | 2 | 2.1 | 2.7 | 0.09 |  |  |
| *qSOC-18-8* | 43946318-44043448 | 3 | 3.4 | 2.6 | 0.11 | 2.3 | 0.09 |
| *qSOC-18-9* | 57497434-57500311 | 3 | 6.2 | 6.7 | 0.28 | 2.7 | 0.11 |
| *qSOC-19-1* | 2265023-2266143 | 3 | 13.7 | 30.4 | 1.29 | 5.9 | 0.25 |
| *qSOC-19-2* | 21726031 | 2 | 5.4 | 4.9 | 0.17 | 3.0 | 0.10 |
| *qSOC-19-3* | 48050361 | 2 | 4.6 | 6.2 | 0.22 |  |  |
| *qSOC-19-4* | 48327403-48405159 | 4 | 6.5 | 2.8 | 0.14 | 8.0 | 0.36 |
| *qSOC-20-1* | 15517239 | 2 | 3.1 | 2.8 | 0.09 | 1.8 | 0.05 |
| *qSOC-20-2* | 19134133-19251740 | 8 | 7.4 | 13.2 | 0.70 |  |  |
| *qSOC-20-3* | 28929188 | 2 | 1.7 | 1.4 | 0.04 | 1.3 | 0.04 |
| *qSOC-20-4* | 33516150-33553942 | 5 | 12.1 | 22.5 | 1.02 | 2.7 | 0.15 |
| *qSOC-20-5* | 43520063-43697026 | 6 | 14.7 | 41.3 | 1.89 | 5.0 | 0.28 |
| **Total** |  | **299** | **92** | **82** | **70.06** | **54** | **9.08** |

A QTL, such as *qSOC-2-1*, is designated in the following way: "SOC" means seed oil content, "-2" represents chromosome 2, and "-1" represents its order (the first) on the chromosome according to position.

AN, number of alleles.

**Table S5 The predicted top 100 crosses for 100-seed weight in NECSGP**

| Order | P1 | MG1 | SW1 | P2 | MG2 | SW2 | P95 | Order | P1 | MG1 | SW1 | P2 | MG2 | SW2 | P95 |
| --- | --- | --- | --- | --- | --- | --- | --- | --- | --- | --- | --- | --- | --- | --- | --- |
| 1 | F364 | 0 | 19.89 | **F67** | III | 25.16 | 30.43 | 51 | F35 | III | 20.78 | F3 | 0 | 22.55 | 28.43 |
| 2 | **F326** | III | 24.28 | F364 | 0 | 19.89 | 30.33 | 52 | F29 | 00 | 21.98 | F35 | III | 20.78 | 28.36 |
| 3 | F309 | 00 | 22.66 | **F67** | III | 25.16 | 29.99 | 53 | F197 | 00 | 20.05 | **F67** | III | 25.16 | 28.35 |
| 4 | **F406** | 0 | 24.15 | **F67** | III | 25.16 | 29.90 | 54 | **F406** | 0 | 24.15 | P087 | I | 21.84 | 28.35 |
| 5 | F35 | III | 20.78 | **P188** | 0 | 23.13 | 29.81 | 55 | **F147** | 0 | 23.93 | F364 | 0 | 19.89 | 28.33 |
| 6 | F35 | III | 20.78 | **F67** | III | 25.16 | 29.54 | 56 | F229 | 0 | 20.54 | F364 | 0 | 19.89 | 28.33 |
| 7 | F364 | 0 | 19.89 | **P188** | 0 | 23.13 | 29.52 | 57 | **F147** | 0 | 23.93 | **F326** | III | 24.28 | 28.31 |
| 8 | **F326** | III | 24.28 | F35 | III | 20.78 | 29.52 | 58 | **F315** | II | 23.05 | **F406** | 0 | 24.15 | 28.28 |
| 9 | F364 | 0 | 19.89 | **F406** | 0 | 24.15 | 29.51 | 59 | F29 | 00 | 21.98 | **F67** | III | 25.16 | 28.26 |
| 10 | F364 | 0 | 19.89 | P345 | 0 | 22.86 | 29.49 | 60 | F364 | 0 | 19.89 | F70 | 0 | 21.53 | 28.26 |
| 11 | F35 | III | 20.78 | P345 | 0 | 22.86 | 29.49 | 61 | F312 | 00 | 22.78 | **F406** | 0 | 24.15 | 28.26 |
| 12 | **F67** | III | 25.16 | P087 | I | 21.84 | 29.46 | 62 | F364 | 0 | 19.89 | **P085** | 0 | 23.59 | 28.25 |
| 13 | F25 | 00 | 22.18 | F35 | III | 20.78 | 29.36 | 63 | F221 | 0 | 20.25 | **F67** | III | 25.16 | 28.24 |
| 14 | **F147** | 0 | 23.93 | **F67** | III | 25.16 | 29.34 | 64 | **F326** | III | 24.28 | P087 | I | 21.84 | 28.23 |
| 15 | F35 | III | 20.78 | **F58** | 000 | 23.84 | 29.27 | 65 | F35 | III | 20.78 | F86 | I | 21.35 | 28.20 |
| 16 | **F315** | II | 23.05 | F364 | 0 | 19.89 | 29.27 | 66 | **F315** | II | 23.05 | **F67** | III | 25.16 | 28.19 |
| 17 | **F58** | 000 | 23.84 | **F67** | III | 25.16 | 29.23 | 67 | F49 | 0 | 20.45 | **F67** | III | 25.16 | 28.19 |
| 18 | **F67** | III | 25.16 | **P085** | 0 | 23.59 | 29.21 | 68 | F35 | III | 20.78 | F80 | 0 | 21.16 | 28.17 |
| 19 | F35 | III | 20.78 | **P085** | 0 | 23.59 | 29.04 | 69 | **F315** | II | 23.05 | **F58** | 000 | 23.84 | 28.15 |
| 20 | **F326** | III | 24.28 | **F67** | III | 25.16 | 28.97 | 70 | F35 | III | 20.78 | F70 | 0 | 21.53 | 28.15 |
| 21 | F364 | 0 | 19.89 | F37 | III | 22.54 | 28.95 | 71 | F28 | 00 | 21.56 | **F67** | III | 25.16 | 28.14 |
| 22 | F35 | III | 20.78 | **F406** | 0 | 24.15 | 28.93 | 72 | P087 | I | 21.84 | P345 | 0 | 22.86 | 28.14 |
| 23 | F35 | III | 20.78 | **P004** | 0 | 23.83 | 28.89 | 73 | F247 | 000 | 20.83 | F35 | III | 20.78 | 28.12 |
| 24 | **F67** | III | 25.16 | **P188** | 0 | 23.13 | 28.88 | 74 | F309 | 00 | 22.66 | **F36** | III | 23.41 | 28.12 |
| 25 | F309 | 00 | 22.66 | **F406** | 0 | 24.15 | 28.87 | 75 | F327 | III | 22.09 | F364 | 0 | 19.89 | 28.11 |
| 26 | **F147** | 0 | 23.93 | F35 | III | 20.78 | 28.85 | 76 | F32 | 0 | 20.94 | F35 | III | 20.78 | 28.11 |
| 27 | **F67** | III | 25.16 | P345 | 0 | 22.86 | 28.84 | 77 | F117 | 0 | 20.75 | F35 | III | 20.78 | 28.11 |
| 28 | F309 | 00 | 22.66 | **F326** | III | 24.28 | 28.83 | 78 | **F147** | 0 | 23.93 | **F315** | II | 23.05 | 28.10 |
| 29 | F364 | 0 | 19.89 | **F36** | III | 23.41 | 28.81 | 79 | F35 | III | 20.78 | F57 | 00 | 22.68 | 28.09 |
| 30 | P087 | I | 21.84 | **P188** | 0 | 23.13 | 28.69 | 80 | F35 | III | 20.78 | F49 | 0 | 20.45 | 28.09 |
| 31 | F35 | III | 20.78 | F61 | 0 | 22.60 | 28.67 | 81 | F35 | III | 20.78 | F45 | 0 | 21.49 | 28.08 |
| 32 | F309 | 00 | 22.66 | **P188** | 0 | 23.13 | 28.66 | 82 | F328 | 0 | 19.65 | **F67** | III | 25.16 | 28.08 |
| 33 | F309 | 00 | 22.66 | **F315** | II | 23.05 | 28.66 | 83 | **F67** | III | 25.16 | F70 | 0 | 21.53 | 28.04 |
| 34 | F312 | 00 | 22.78 | F364 | 0 | 19.89 | 28.65 | 84 | F309 | 00 | 22.66 | F37 | III | 22.54 | 28.03 |
| 35 | F312 | 00 | 22.78 | F35 | III | 20.78 | 28.64 | 85 | F313 | 000 | 22.18 | **F67** | III | 25.16 | 28.03 |
| 36 | F61 | 0 | 22.60 | **F67** | III | 25.16 | 28.62 | 86 | F57 | 00 | 22.68 | **F67** | III | 25.16 | 28.03 |
| 37 | F25 | 00 | 22.18 | F364 | 0 | 19.89 | 28.62 | 87 | **F315** | II | 23.05 | **F326** | III | 24.28 | 28.03 |
| 38 | F364 | 0 | 19.89 | **F58** | 000 | 23.84 | 28.62 | 88 | **F315** | II | 23.05 | F35 | III | 20.78 | 28.02 |
| 39 | **F67** | III | 25.16 | F80 | 0 | 21.16 | 28.61 | 89 | F25 | 00 | 22.18 | **F67** | III | 25.16 | 28.02 |
| 40 | **F67** | III | 25.16 | **P004** | 0 | 23.83 | 28.59 | 90 | **F315** | II | 23.05 | P087 | I | 21.84 | 28.00 |
| 41 | F364 | 0 | 19.89 | **P004** | 0 | 23.83 | 28.58 | 91 | F364 | 0 | 19.89 | F65 | III | 20.20 | 27.99 |
| 42 | F248 | III | 22.94 | F35 | III | 20.78 | 28.57 | 92 | F313 | 000 | 22.18 | F364 | 0 | 19.89 | 27.99 |
| 43 | F221 | 0 | 20.25 | F364 | 0 | 19.89 | 28.57 | 93 | F3 | 0 | 22.55 | **F67** | III | 25.16 | 27.98 |
| 44 | F248 | III | 22.94 | F364 | 0 | 19.89 | 28.56 | 94 | F364 | 0 | 19.89 | F3 | 0 | 22.55 | 27.98 |
| 45 | F182 | 00 | 21.84 | F35 | III | 20.78 | 28.56 | 95 | **F58** | 000 | 23.84 | P087 | I | 21.84 | 27.97 |
| 46 | F35 | III | 20.78 | F63 | III | 22.01 | 28.55 | 96 | F286 | 0 | 21.98 | **F67** | III | 25.16 | 27.97 |
| 47 | F182 | 00 | 21.84 | **F67** | III | 25.16 | 28.54 | 97 | F28 | 00 | 21.56 | F35 | III | 20.78 | 27.96 |
| 48 | **F147** | 0 | 23.93 | **F406** | 0 | 24.15 | 28.47 | 98 | F364 | 0 | 19.89 | F63 | III | 22.01 | 27.96 |
| 49 | F248 | III | 22.94 | **F67** | III | 25.16 | 28.44 | 99 | **F326** | III | 24.28 | F327 | III | 22.09 | 27.95 |
| 50 | F309 | 00 | 22.66 | P345 | 0 | 22.86 | 28.43 | 100 | F35 | III | 20.78 | **F36** | III | 23.41 | 27.95 |

P1 and P2 are the abbreviation for parent 1 and 2. ID: accession name. MG: maturity group. 100SW: 100-seed weight (g). P95: the 95th percentile. Accession name in boldface represents the top10 100SW accessions, which are F67, F326, F406, F147, F58, P004, P085, F36, P188 and F315 in descending order in the NECSGP.

**Table S6 The predicted top 100 crosses for seed oil content in NECSGP**

| Order | P1 | MG1 | SOC1 | P2 | MG2 | SOC2 | P95 | Order | P1 | MG1 | SOC1 | P2 | MG2 | SOC2 | P95 |
| --- | --- | --- | --- | --- | --- | --- | --- | --- | --- | --- | --- | --- | --- | --- | --- |
| 1 | F140 | 00 | 23.88 | F344 | II | 24.01 | 27.73 | 51 | F104 | II | 22.25 | F343 | II | 23.45 | 26.76 |
| 2 | F140 | 00 | 23.88 | F343 | II | 23.45 | 27.54 | 52 | F109 | II | 24.00 | F96 | I | 23.79 | 26.75 |
| 3 | F140 | 00 | 23.88 | F96 | I | 23.79 | 27.48 | 53 | **F306** | 0 | 24.10 | **F82** | 0 | 24.73 | 26.75 |
| 4 | F109 | II | 24.00 | **F82** | 0 | 24.73 | 27.42 | 54 | F109 | II | 24.00 | F55 | 0 | 23.60 | 26.74 |
| 5 | F109 | II | 24.00 | **F306** | 0 | 24.10 | 27.29 | 55 | F109 | II | 24.00 | F141 | 000 | 23.79 | 26.73 |
| 6 | F109 | II | 24.00 | **F32** | 0 | 24.31 | 27.28 | 56 | **F82** | 0 | 24.73 | F96 | I | 23.79 | 26.73 |
| 7 | F343 | II | 23.45 | **F82** | 0 | 24.73 | 27.27 | 57 | F343 | II | 23.45 | **F53** | 0 | 24.19 | 26.72 |
| 8 | F109 | II | 24.00 | F140 | 00 | 23.88 | 27.27 | 58 | **F135** | 0 | 24.43 | F343 | II | 23.45 | 26.72 |
| 9 | F344 | II | 24.01 | **F386** | 0 | 24.54 | 27.26 | 59 | **F305** | 0 | 24.16 | F83 | 0 | 23.96 | 26.72 |
| 10 | **F32** | 0 | 24.31 | F344 | II | 24.01 | 27.23 | 60 | F148 | 00 | 24.05 | **F82** | 0 | 24.73 | 26.72 |
| 11 | F344 | II | 24.01 | **F82** | 0 | 24.73 | 27.23 | 61 | **F305** | 0 | 24.16 | F343 | II | 23.45 | 26.71 |
| 12 | F110 | I | 23.20 | F140 | 00 | 23.88 | 27.17 | 62 | F109 | II | 24.00 | F320 | 00 | 23.39 | 26.71 |
| 13 | F109 | II | 24.00 | F344 | II | 24.01 | 27.14 | 63 | F148 | 00 | 24.05 | F83 | 0 | 23.96 | 26.71 |
| 14 | **F386** | 0 | 24.54 | F83 | 0 | 23.96 | 27.12 | 64 | F244 | 00 | 23.70 | F343 | II | 23.45 | 26.71 |
| 15 | **F32** | 0 | 24.31 | F343 | II | 23.45 | 27.11 | 65 | F109 | II | 24.00 | F250 | 00 | 23.65 | 26.70 |
| 16 | F140 | 00 | 23.88 | F83 | 0 | 23.96 | 27.10 | 66 | **F79** | 0 | 24.33 | **F82** | 0 | 24.73 | 26.70 |
| 17 | **F386** | 0 | 24.54 | **F82** | 0 | 24.73 | 27.10 | 67 | F109 | II | 24.00 | F22 | I | 23.60 | 26.69 |
| 18 | F109 | II | 24.00 | F83 | 0 | 23.96 | 27.07 | 68 | **F306** | 0 | 24.10 | F83 | 0 | 23.96 | 26.69 |
| 19 | F109 | II | 24.00 | **F305** | 0 | 24.16 | 27.06 | 69 | F109 | II | 24.00 | F166 | 00 | 22.34 | 26.69 |
| 20 | F109 | II | 24.00 | F244 | 00 | 23.70 | 27.01 | 70 | **F306** | 0 | 24.10 | **F32** | 0 | 24.31 | 26.69 |
| 21 | F109 | II | 24.00 | F148 | 00 | 24.05 | 27.01 | 71 | F109 | II | 24.00 | F226 | I | 23.43 | 26.68 |
| 22 | **F32** | 0 | 24.31 | F96 | I | 23.79 | 27.01 | 72 | F136 | 0 | 23.58 | F344 | II | 24.01 | 26.68 |
| 23 | F109 | II | 24.00 | **F53** | 0 | 24.19 | 26.99 | 73 | **F82** | 0 | 24.73 | F84 | 0 | 23.23 | 26.68 |
| 24 | F136 | 0 | 23.58 | **F386** | 0 | 24.54 | 26.98 | 74 | **F305** | 0 | 24.16 | F344 | II | 24.01 | 26.67 |
| 25 | F344 | II | 24.01 | **F53** | 0 | 24.19 | 26.98 | 75 | **F351** | 0 | 24.09 | **F82** | 0 | 24.73 | 26.67 |
| 26 | **F32** | 0 | 24.31 | F83 | 0 | 23.96 | 26.98 | 76 | F109 | II | 24.00 | F343 | II | 23.45 | 26.67 |
| 27 | F104 | II | 22.25 | F344 | II | 24.01 | 26.97 | 77 | **F53** | 0 | 24.19 | **F82** | 0 | 24.73 | 26.67 |
| 28 | F110 | I | 23.20 | **F82** | 0 | 24.73 | 26.97 | 78 | F136 | 0 | 23.58 | F343 | II | 23.45 | 26.66 |
| 29 | F110 | I | 23.20 | **F386** | 0 | 24.54 | 26.94 | 79 | F244 | 00 | 23.70 | F83 | 0 | 23.96 | 26.66 |
| 30 | F343 | II | 23.45 | **F386** | 0 | 24.54 | 26.93 | 80 | F110 | I | 23.20 | **F32** | 0 | 24.31 | 26.66 |
| 31 | **F306** | 0 | 24.10 | F344 | II | 24.01 | 26.92 | 81 | F109 | II | 24.00 | F374 | 0 | 23.03 | 26.66 |
| 32 | **F155** | 0 | 24.28 | **F82** | 0 | 24.73 | 26.91 | 82 | **F305** | 0 | 24.16 | **F82** | 0 | 24.73 | 26.66 |
| 33 | **F32** | 0 | 24.31 | **F386** | 0 | 24.54 | 26.91 | 83 | F140 | 00 | 23.88 | **F79** | 0 | 24.33 | 26.65 |
| 34 | **F135** | 0 | 24.43 | F344 | II | 24.01 | 26.90 | 84 | **F306** | 0 | 24.10 | **F386** | 0 | 24.54 | 26.65 |
| 35 | F140 | 00 | 23.88 | **F386** | 0 | 24.54 | 26.89 | 85 | F109 | II | 24.00 | F206 | 0 | 23.64 | 26.65 |
| 36 | F140 | 00 | 23.88 | **F82** | 0 | 24.73 | 26.86 | 86 | F344 | II | 24.01 | F88 | 0 | 23.25 | 26.65 |
| 37 | F109 | II | 24.00 | **F155** | 0 | 24.28 | 26.86 | 87 | **F386** | 0 | 24.54 | F68 | 0 | 23.33 | 26.64 |
| 38 | F244 | 00 | 23.70 | F344 | II | 24.01 | 26.85 | 88 | **F155** | 0 | 24.28 | **F386** | 0 | 24.54 | 26.64 |
| 39 | F109 | II | 24.00 | **F135** | 0 | 24.43 | 26.85 | 89 | F206 | 0 | 23.64 | F343 | II | 23.45 | 26.64 |
| 40 | **F53** | 0 | 24.19 | F96 | I | 23.79 | 26.85 | 90 | **F351** | 0 | 24.09 | F96 | I | 23.79 | 26.63 |
| 41 | F109 | II | 24.00 | F84 | 0 | 23.23 | 26.84 | 91 | F321 | 00 | 23.53 | **F82** | 0 | 24.73 | 26.63 |
| 42 | **F386** | 0 | 24.54 | F84 | 0 | 23.23 | 26.84 | 92 | F206 | 0 | 23.64 | **F82** | 0 | 24.73 | 26.62 |
| 43 | **F32** | 0 | 24.31 | **F82** | 0 | 24.73 | 26.84 | 93 | F109 | II | 24.00 | F313 | 000 | 23.05 | 26.62 |
| 44 | **F386** | 0 | 24.54 | F96 | I | 23.79 | 26.83 | 94 | F109 | II | 24.00 | F110 | I | 23.20 | 26.62 |
| 45 | **F53** | 0 | 24.19 | F83 | 0 | 23.96 | 26.81 | 95 | F109 | II | 24.00 | **F386** | 0 | 24.54 | 26.62 |
| 46 | F109 | II | 24.00 | F136 | 0 | 23.58 | 26.80 | 96 | F110 | I | 23.20 | **F53** | 0 | 24.19 | 26.61 |
| 47 | **F386** | 0 | 24.54 | F49 | 0 | 23.06 | 26.80 | 97 | F100 | I | 23.58 | F140 | 00 | 23.88 | 26.60 |
| 48 | **F135** | 0 | 24.43 | F96 | I | 23.79 | 26.79 | 98 | **F305** | 0 | 24.16 | **F32** | 0 | 24.31 | 26.60 |
| 49 | **F82** | 0 | 24.73 | F83 | 0 | 23.96 | 26.77 | 99 | F136 | 0 | 23.58 | F96 | I | 23.79 | 26.60 |
| 50 | **F386** | 0 | 24.54 | **F79** | 0 | 24.33 | 26.77 | 100 | F148 | 00 | 24.05 | F344 | II | 24.01 | 26.60 |

P1 and P2 are the abbreviation for parent 1 and 2. ID: accession name. MG: maturity group. SOC: seed oil content (%). P95: the 95^th^ percentile. Accession name in boldface represents the top10 SOC accessions, which are F82, F386, F135, F79, F32, F155, F53, F305, F306 and F351 in descending order in the NECSGP.
